# Recent tree cover increases in eastern China linked to low, declining human pressure, steep topography, and climatic conditions favoring tree growth

**DOI:** 10.1371/journal.pone.0177552

**Published:** 2017-06-07

**Authors:** Jonas Nüchel, Jens-Christian Svenning

**Affiliations:** 1 Section for Ecoinformatics & Biodiversity, Department of Bioscience, Aarhus University, Aarhus, Denmark; 2 Sino-Danish Center for Education and Research, Beijing, China; Chinese Academy of Forestry, CHINA

## Abstract

Globally, the extent of forest continues to decline, however, some countries have increased their forest extent in recent years. China is one of these countries and has managed to increase their tree cover through huge reforestation and afforestation programs during recent decades as well as land abandonment dynamics. This study investigates tree cover change in the eastern half of China between 2000 and 2010 on three different scales, using random forest modeling of remote sensing data for tree cover in relation to environmental and anthropogenic predictor variables. Our results show that between the years 2000 and 2010 2,667,875 km^2^ experienced an increase in tree cover while 1,854,900 km^2^ experienced a decline in tree cover. The area experiencing ≥10% increase in tree cover is almost twice as large as the area with ≥10% drop in tree cover. There is a clear relation between topography and tree cover change with steeper and mid-elevation areas having a larger response on tree cover increase than other areas. Furthermore, human influence, change in population density, and actual evapotranspiration are also important factors in explaining where tree cover has changed. This study adds to the understanding of tree cover change in China, as it has focus on the entire eastern half of China on three different scales and how tree cover change is linked to topography and anthropogenic pressure. Though, our results show an increase in tree cover in China, this study emphasizes the importance of incorporating anthropogenic factors together with biodiversity protection into the reforestation and afforestation programs in the future.

## 1. Introduction

Globally, the extent of forest continues to decline and since 1990 more than 1,290,000 km^2^ of forest has disappeared [1,2]. In recent years, the decline has slowed and some countries, mainly developed northern countries, has even increased there tree cover in recent decades [1,2]. However, deforestation of natural forest and forest in the tropics continues [2–4].

The change in tree cover is of great concern, as forests are the primary habitat type for terrestrial biodiversity and deforestation is one of the main drivers of biodiversity loss [5,6]. Furthermore, forests offer many ecosystems services for humans, such as soil and flood protection, food, recreational uses, wood products, aesthetic and spiritual values, climate control, e.g. exchanges of water and carbon dioxide, storage of carbon etc. [7–11]. Nevertheless, forests have undergone drastic transformation during recent time and forests in China are no exception.

China has a long history of anthropogenic impact, but has experienced unprecedented population growth during the 20^th^ century and substantial economic growth during recent decades [12]. These changes have had a big impact on the forests in China and huge areas have been deforested and forest age and composition has changed substantially [12–14]. For example during 1960s and 1970s where a national strategy of food self-sufficiency made farming on steep slopes more widespread, with deforestation of sloped terrain as a consequence [14]. Usually, sloped terrain and higher elevation in general have a certain natural protection for deforestation because of its difficult accessibility to human activity with e.g. agriculture, and studies have shown that there is a connection between topography and tree cover [15–17].

These large impacts on forests in China, however, have led to a growing recognition of the importance of forests and the need to protect them. In addition to an extensively increase in the number of protected areas during the last decades [18,19], China has launched several programs to protect and increase the tree cover within its borders. The first huge program was the Three North Shelterbelt Program, which was an afforestation program, launched in 1978 to prevent or reduce further desertification [20]. In 1998 the Natural Forest Protection Program was launched and in1999 the Grain for Green Program (Also known as Slope Land Conversion Program or the Conversion of Cropland to Forest Program), the latter first locally launched but in 2001 it became national. These programs increased the afforestation and reforestation substantially [14,21–23] and have led to an overall increase in tree cover in China [1]. In recent decades China has also experienced massive rural to urban migration and as a consequence land abandonment, especially in areas that are less attractive for agriculture, e.g. sloped terrain is being abandoned [24–26].

Other studies have investigated patterns of tree cover and tree cover changes locally in China; however, none have focused on how tree cover changes are linked to anthropogenic, climatic, and topographic factors in the entire eastern half of China. The aim of this study is to investigate how tree cover has changed in the eastern half of China between 2000 and 2010. We hypothesize that tree cover overall has increased and mainly in areas that have experienced a population decline and has low human influence. Furthermore, we hypothesize that increased actual evapotranspiration and topographic slope has a positive effect on tree cover change. Lastly, we also investigate if tree cover has increased especially within protected areas.

## 2. Materials and methods

### 2.1 Study area

**Fig 1:**
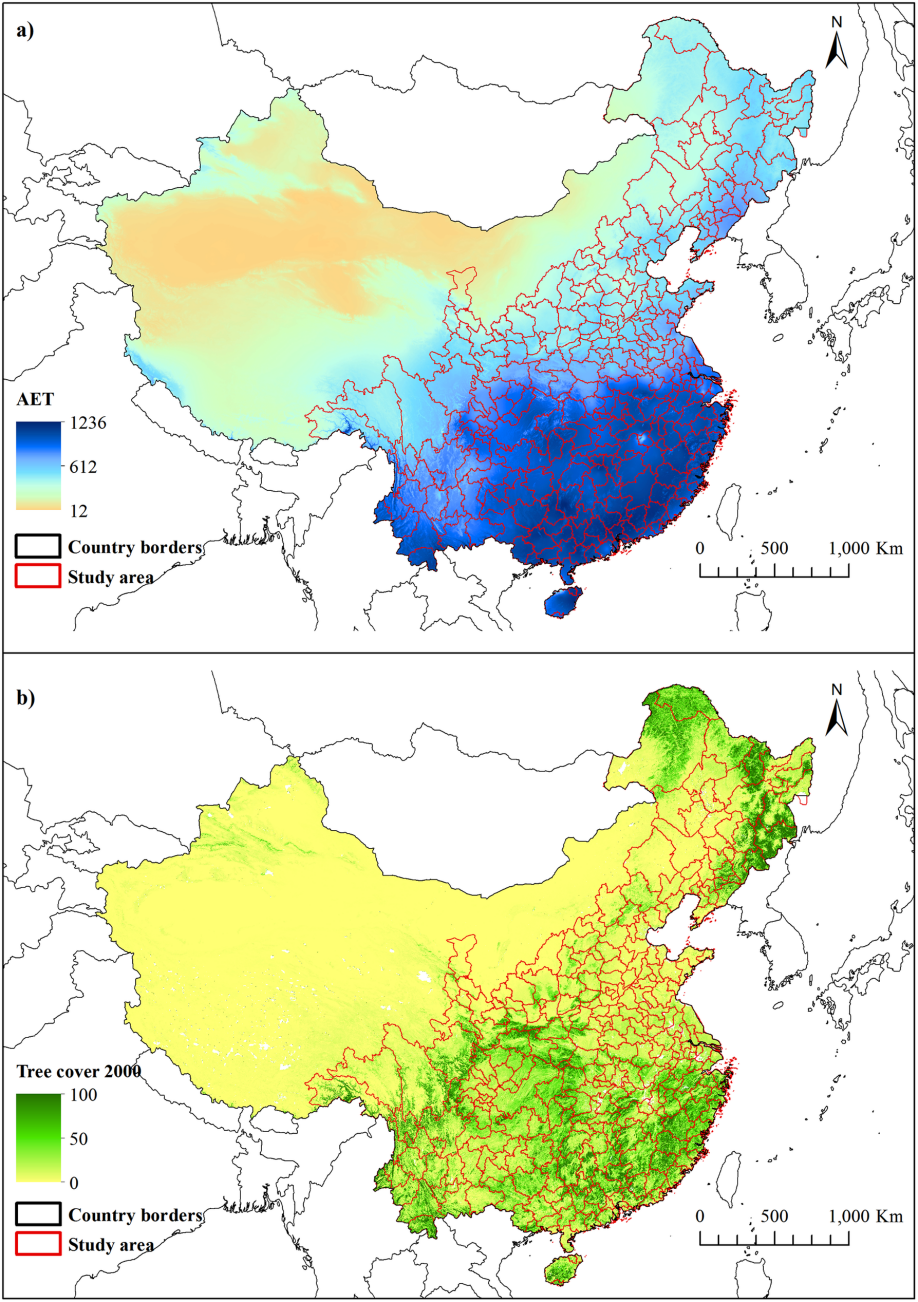
**Study area and a) actual evapotranspiration for China and b) Study area and tree cover 2000 for China**. See supporting information S1 Fig for tree cover 2010 for China.

Our study encompasses a vast area (4.873.475 km^2^) located in China, which roughly corresponds to the eastern half of China (Fig 1). The area was chosen by selecting all prefectures in China were half or more of the counties within had more than 400 mm annual precipitation. This included 300 prefectures, 2086 counties, and 194.939 5×5 km grid cells all located in the eastern part of China. China is a huge country with diverse climate, but in general eastern China has a wetter climate and thus naturally more tree cover than western China. The excluded parts of China were all of Xinjiang Uyghur Autonomous Region, most of Tibet Autonomous Region, Qinghai province and Gansu province, and approximately half of Ningxia Autonomous Region and Inner Mongolia Autonomous Region.

### 2.2 Environmental and anthropogenic data

We used tree cover data for 11 years between 2000 and 2010 from the Moderate Resolution Imaging Spectroradiometer (MODIS) Vegetation Continuous Fields (VCF) dataset (250 m resolution) [27] and calculated overall tree cover change and the per-year change rate from 2000 to 2010. The per-year change rate for each cell was calculated by fitting a linear regression between year and tree cover for all cells in each year between 2000 and 2010. The slope of the regression was taken to be the tree cover change rate. MODIS VCF has been validated and found to overall perform well [27–30]. However, studies have found that MODIS VCF shows uncertainty in estimating tree cover and tree cover changes in semi-arid areas [31].

For topographic data we used elevation data (supporting information S2A Fig) derived from Shuttle Radar Topography Mission (90 m resolution) [32] and calculated slope (supporting information S2B Fig). Regarding climate data more specifically water availability in relation to energy input, we used mean annual actual evapotranspiration (AET) from the Global-High-Resolution Soil-Water Balance dataset (30 arcsec resolution) [33] (supporting information S2C Fig).

Human population density, Human Influence Index (HII) and Gross Domestic Product (GDP) were used as variables to represent anthropogenic activities. For human population density we used the years 2000 (PopD2000) and 2010 (PopD2010) (30 arcsec resolution) [34] and calculated change in population density between 2000 and 2010 (PC00-10) (supporting information S2D Fig). Human Influence Index (HII) (1 km resolution) [35] (supporting information S2E Fig), which is an index going from 0 (no impact) to 64 (maximum impact), combines data for population density with data for human land use and accessibility (roads, railroads, navigable rivers and coastlines) and can be used to describe anthropogenic impacts on the environment. Gross domestic product (GDP) for the year 2000 for all counties in China [36] were obtained and we calculated the GDP per area (km^2^) for the counties (supporting information S2F Fig) and prefectures.

In addition, we derived data for protected areas from World Database on Protected Areas [37] and made a 10 km buffer around all the protected areas and then calculated whether the majority of a 5×5 km grid cell were within a protected area, in a buffer, or outside the protected areas.

All data were projected to the Albers Equal Area Conic projection and converted to their mean values for 5×5 km grid cells, counties and prefectures. We used ArcGIS 10.2 (ESRI, Redlands, CA) for all GIS operations.

Additionally, we calculated Pairwise Pearson’s correlation coefficient (r) for all variables on all scales (supporting information S1 Table). Tree cover change between 2000 and 2010 (TCC) was correlated with the per-year change rate (CR) on all scales (r>0.7). See supporting information S3 Fig for comparison between TCC and CR on all scales.

### 2.3 Random Forest

To determine which environmental and anthropogenic variables that best explained the tree cover changes between 2000 and 2010 (TCC) we used the R package randomForest v. 4.6–12 [38] and ran Random Forest regression [39] on the three scales; 5×5 km grid cells, counties and prefectures. On the 5×5 km grid cell scale we ran three different analyses; one with all cells, one with all cells experiencing a change (increase or decrease) of 10% or more, and one with all cells experiencing a change (increase or decrease) of 15% or more. The latter two were incorporated to get a more robust result and leave out cells with small changes that might be wrongly classified as cells having an increase or decrease in tree cover.

Random Forest (RF) is used to assess variable importance and predict species distribution. It is a machine learning technique that ensembles many regression and classification trees, thereby the forest, to reduce the variance and improve prediction accuracy. The randomness in the “Forest” appears because each tree is based on a random subset of the observations (bootstrap samples) and that each split in each tree is based on a random subset of variables. RF is among the best performing machine learning models for regression and classification regarding variable importance and prediction of species distribution [40–42].

The performance of the RF models may be influenced by the number of variables in each split (mtry) and the number of trees (ntree) in the “forest”. However, changes in ntree and mtry in most cases have negligible effects [43]. Here, we tried with a different number of variables in each split, but found that the default value for mtry (number of variables divided by 3) yielded optimal or close to optimal performance of all the models. We also tried different ntree values and found that the models only improved slightly above 100 trees (supporting information S4 Fig). As computation time increased with increasing ntree, we therefore used ntree = 1.000 in our modelling.

Correlated variables do not influence the predictive power of RF [39], but may affect estimated variable importance as they might mask the importance of each other. Consequently, we kept correlated variables (r > 0.7, see supporting information S1 Table) in separate models on all scales. For example, population densities for 2000 (PopD2000), 2010 (PopD2010) and the change between 2000–2010 (PC00-10) were, not surprisingly, correlated with each other (supporting information S1 Table), and they were therefore only included in separate models (Table 1). As population density in the various years and the change between the years had similar explanatory power (±0.5% variance explained), we used the change between 2000 and 2010 in the analyses presented here.

**Table 1:** Comparison of selected random forest models with increased complexity for the three scales.

Scale	MSR	%Var. explained	Variables (after importance)^a^				
5×5 km grid cells	202.3	8.3	Elevation***	Slope***	AET***	PC00-10***	HII***
Counties	10.8	47.2	Slope***	Elevation***	GDP/Area***	AET***	PC00-10***
Prefectures	8.0	41.0	Slope***	Elevation***	AET***	PC00-10***	


^a^ Variables listed according to their importance (see results section).

*** = p-value ≤ 0.005.

Acronyms: MSR = mean of squared residuals (error), %Var. explained = percentage of variance the random forest models can explain (explanatory power), AET = actual evapotranspiration, GDP/Area = gross domestic product per km^2^, PC00-10 = Change in population density between 2000 and 2010, HII = Human Influence Index.

We used the permutation-based mean squared error (MSE) reduction [43,44] and the permutation importance (PIMP) algorithm [45] in R package vita (Variable Importance Testing Approaches) version 1 [46] to investigate which variables were important in the models. Additionally, we used recursive feature elimination [47], where the least relevant variable is eliminated and a new permutation importance measure is computed at each step to find the simplest model with the most relevant variables.

## 3. Results

**Fig 2:**
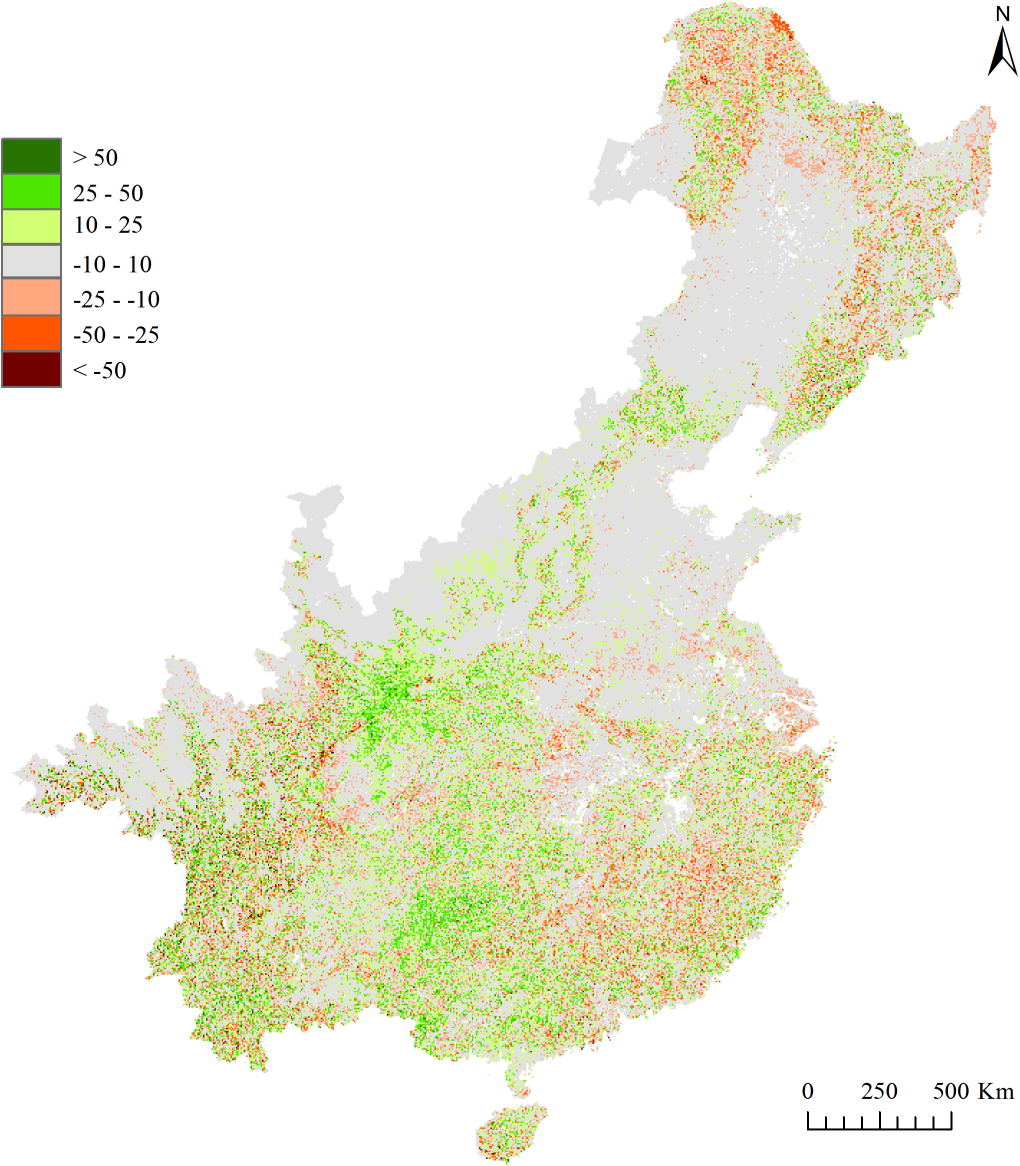
Tree cover change in percent between 2000 and 2010 (TCC) for 5×5 km grid cells. Green colors indicate an increase, gray colors indicate a slight increase or decrease and red colors indicate a decrease in tree cover between 2000 and 2010. See supporting information S5 Fig for tree cover change (TCC) on county and prefecture scale.

Overall, 2,667,875 km^2^ of the eastern China study area has experienced an increase in tree cover between 2000 and 2010, hereof 1,165,250 km^2^ with an increase of ≥ 10% tree cover change. In contrast, 1,854,900 km^2^ of the eastern China study area has experienced a mean decrease in tree cover, herof 608,450 km^2^ with a mean decrease ≤ -10% tree cover change (Fig 2). On the county and prefecture scale, there are 1587 counties and 229 prefectures that has experienced a mean increase in tree cover and 554 counties and 71 prefectures that has experienced a mean decrease in tree cover (supporting information S5 Fig).

Some areas in eastern China have experienced particularly strong tree cover changes. Tree cover increases have been strongest in the central parts, mainly in east Sichuan, southeast Guizhou, south Gansu and Shaanxi, west Hubei and Hunan, southwest Yunnan and in Guangxi. In contrast, tree cover losses have mainly occurred in the northern and eastern parts of eastern China, but also in central and south Sichuan (Fig 2 and supporting information S5 Fig).

The random forest (RF) models did well on county and prefecture scale where the best models explained 47.2% and 41.0% of tree cover change variation. Conversely, the RF model on the 5×5 km grid cell scale only explained 8.3% of tree cover change variation. However, the performance increased when we only included cells with a substantial increase or decrease in tree cover. The RF models on the 5×5 km grid cell scale, using only cells that had experienced an increase or decrease of 10% or more or of 15% or more, explained 11.5% and 13.6% of the variation, respectively.

On all three scales slope, elevation, actual evapotranspiration (AET) and population density change between 2000 and 2010 (PC00-10) were important explanatory factors for tree cover changes (Table 1). Human Influence Index (HII) were also important at the 5×5 km scale (Table 1), but not on the coarser scales. In contrast, Gross Domestic Product per area (GDP/Area) was important at the county scale (Table 1). Whether a grid cell was assigned to be inside a protected area, in a buffer, or fully outside it never significantly contributed to the model’s explanatory power and was thus left out of the final models. The variable importance rank did not change for the RF models on the 5×5 km grid cell scale regardless of all cells were included or only cells experiencing a change of 10% or 15% or more were included.

**Fig 3:**
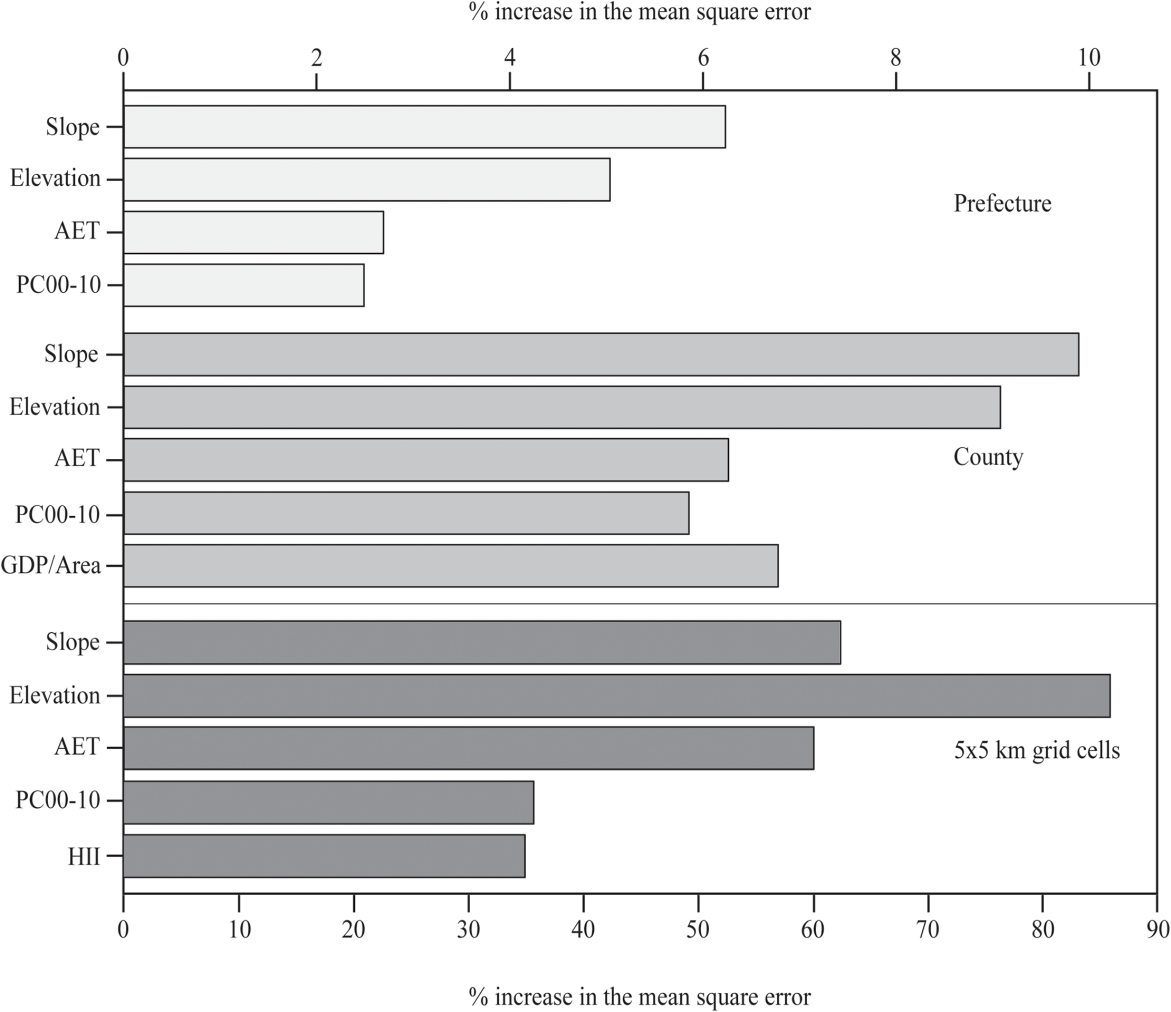
Variable importance for the random forest models for the county and prefecture scale (top axis) and the 5×5 km grid cells scale (bottom axis). The variable importance is calculated by comparing the mean squared error from models with the original dataset with the mean squared error from models with an altered dataset where the predictor variable is randomly permuted. Acronyms: AET = actual evapotranspiration, PC00-10 = Change in population density between 2000 and 2010, GDP/Area = gross domestic product per km^2^, HII = Human Influence Index.

The importance rank of the explanatory variables was largely consistent between scales, with only small shifts. Slope and elevation always constituted the two most important variables (Fig 3). Furthermore, AET was also always of higher importance than PC00-10 (Fig 3). However, GDP/Area was more important than both at the county scale (Fig 3).

**Fig 4:**
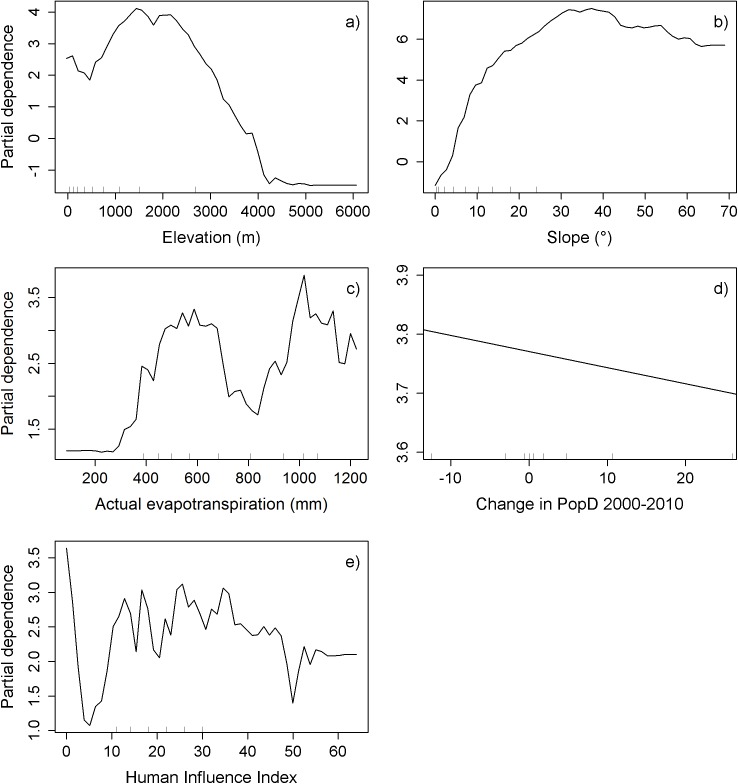
Partial dependence plots of the variables in the random forest model on the 5×5 km grid cells scale. **a)** slope, **b)** elevation, **c)** actual evapotranspiration, **d)** change in population density between 2000 and 2010, and **e)** Human Influence Index. The ticks inside the graphs indicate the deciles for the data. The x-axis in partial dependence plot **d)** has been cut off at the 1^st^ and 9^th^ decile, so the graph only shows the mid 80 percent of the data. See supporting information S6B Fig for the complete partial dependence plot of change in population density between 2000 and 2010.

**Fig 5:**
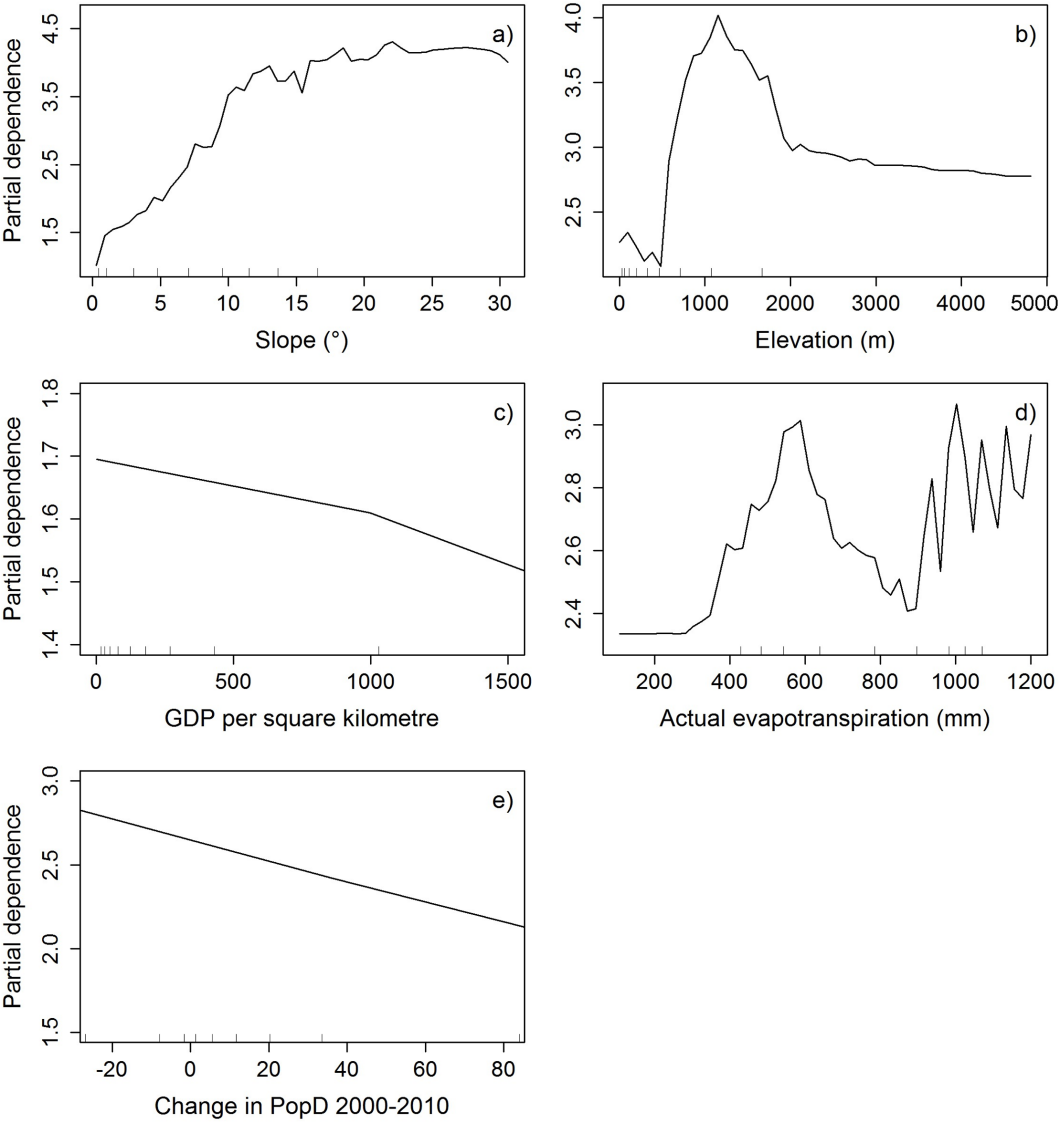
Partial dependence plots of the variables in the random forest model on county scale. **a)** slope, **b)** elevation, **c)** GDP per square kilometer, **d)** actual evapotranspiration, and **e)** change in population density between 2000 and 2010. The ticks inside the graphs indicate the deciles for the data. The x-axis in partial dependence plot **c)** has been cut off so big outliners above the 9^th^ decile are not shown. See supporting information S6D Fig for the complete partial dependence plot of GDP per square kilometer. The x-axis in partial dependence plot **e)** has been cut off at the 1^st^ and 9^th^ decile, so the graph only shows the mid 80 percent of the data. See supporting information S6C Fig for the complete partial dependence plot of change in population density between 2000 and 2010.

**Fig 6:**
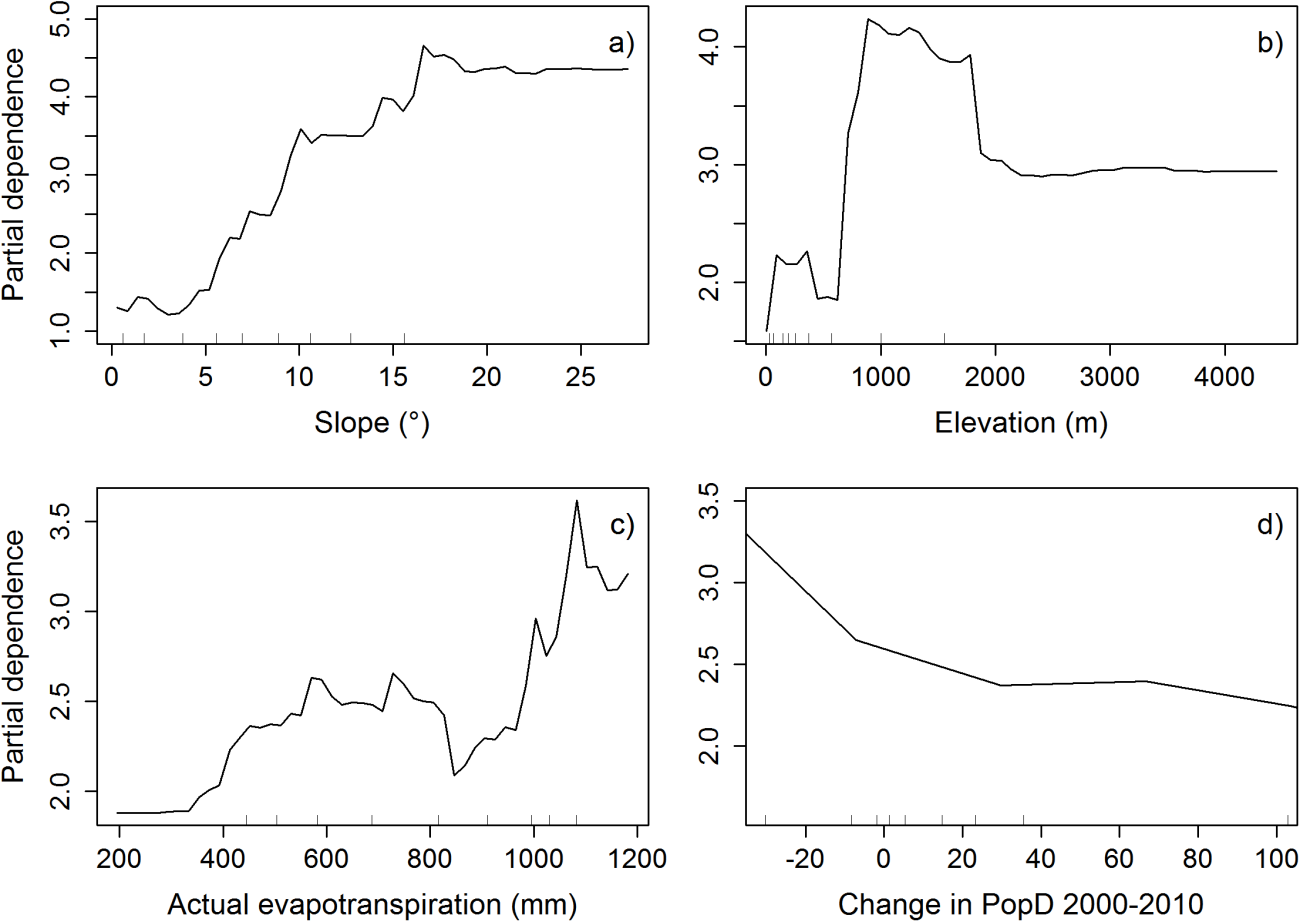
Partial dependence plots of the variables in the random forest model on prefecture scale. **a)** slope, **b)** elevation, **c)** actual evapotranspiration, and **d)** change in population density between 2000 and 2010. The ticks inside the graphs indicate the deciles for the data. The x-axis in partial dependence plot **d)** has been cut off at the 1^st^ and 9^th^ decile, so the graph only shows the mid 80 percent of the data. See supporting information S6A Fig for the complete partial dependence plot of change in population density between 2000 and 2010.

The marginal response of slope increases as steepness increases (Figs 4B, 5A and 6A) and the effect of elevation increases with higher elevation until the effect again reduces and levels off (Figs 4A, 5B and 6B). The importance of AET on TCC overall increases with increasing AET, but fluctuates at the higher values (Figs 4C, 5C and 6C). Changes in population density are most important (the marginal response of TCC is highest) when they are negative and the importance decreases with increasing population density changes (Figs 4D, 5E and 6D). The data for change in population density had a few data points with huge values, but was mainly concentrated around zero. Therefore, the axes in Figs 4D, 5E and 6D are cut off to show only the mid 80% of the data. See supporting information S6A Fig, S6C Fig and S6B Fig for the full partial dependence plots. In the top 10% of the data the effect seems to increase some again and then levels off as population density change increases (supporting information S6A, S6C and S6B Fig). However, the few data points for the very high population density changes make it difficult to interpret the effect of these on tree cover changes. The same applies to the GDP/Area data, which also have very few huge values. For the majority of data the marginal response decreases with increasing GDP/Area (Fig 5C), but increases with the very few and very high values (supporting information S6D Fig). The marginal response of HII fluctuates a lot but overall shows a trend with decreasing response as HII increases (Fig 4E).

The importance and the response of the variables also correspond to those areas which experienced a tree cover increase between 2000 and 2010 and are in general located in areas with higher slopes, mid elevations between 1000–2000 m, human population declines or small increases, and low to moderate GDP/Area (supporting information S7, S8 and S9 Figs).

## 4. Discussion

There has been both increase and decrease in tree cover throughout the study area, but overall increase predominates and especially in central parts of eastern China. Between 2000 and 2010 2,667,875 km^2^ in the eastern half of China experienced a tree cover increase, while 1,854,900 km^2^ experienced a tree cover decline. 1,165,250 km^2^ have had an increase of ≥ 10%, almost twice the area that have had a decrease of ≤ -10%. Slope, elevation, actual evapotranspiration (AET) and change in population density between 2000–2010 (PC00-10) were all important in explaining the tree cover changes. Human Influence Index (HII) and GDP per area (GDP/Area) were also important factors on the 5×5 km scale and the county scale, respectively. Overall, these findings show that recent tree cover increase in eastern China is associated with low and declining human pressure, as well as steep terrain with limited utility for human activities and climatic conditions favoring tree growth. These relations will be discussed in the following.

The marginal response of TCC in relation to Human Influence Index (HII) fluctuates a lot on the fine scale, but overall decreases as HII increases. The reason for the fluctuation could be that some areas with low HII already have relatively high tree cover, or other areas have climatic conditions which neither favors tree cover or anthropogenic presence. Another aspect is that in many cases areas with high HII have formerly been extensively deforested and are therefore only capable of experiencing an increase in tree cover, especially with the growing awareness of the importance of tree cover.

The marginal response of TCC in relation to changes in population density (PC00-10) was highest when the PC00-10 was negative and overall decreases as PC00-10 increased positively. Many of the areas that have experienced a decline in population density and an increase in tree cover are likely areas with marginal farmlands which have been abandoned—especially in areas with steep slopes, due to rural to urban migration [25]. The Slope Land Conversion program has also particularly targeted marginal farmland with slopes over 25° for reforestation and afforestation [14,22,23,48], which likely has influenced the relation between TCC and topography.

We also see a relation between TCC and topography as the marginal response of TCC increases as slope increases and at elevations between approximately 500 m and 2000–2500 m. Slope and elevation are linked to each other, as it is usually steepest at mid elevation on a mountain. Low elevation and flat terrain are most attractive for anthropogenic use and a number of other studies have also found a connection between topography and tree cover [15–17,49] or between topography and protected areas [50–52], all due to anthropogenic factors. This anthropogenic preference for areas in low elevation and with low slopes can also be seen in supporting information S10 Fig, which shows a clear trend on all scales with decreasing human influence as slope and elevation increases. The decrease of TCC response on elevation after approximately 1500–2500 m could also be caused by the relation between elevation and slope, as mountains often flattens out near the top and these areas again become attractive to anthropogenic use, e.g. as pastures for livestock. However, it is probably also caused by climatic conditions that do not favor tree cover, e.g. some of the high elevations are above the tree line and some are on the Tibetan plateau were it is too dry.

The relationship between AET and TCC is probably also influenced by anthropogenic factors. Not surprisingly, the response of TCC is low, when AET is low. Above approximately 300 mm the marginal response of TCC begins to increase and there is an overall positive relation between increase in AET and increase in the response of TCC. However, the response drops again around 700 mm and there is no natural explanation to this drop. A possible explanation could be that as AET increases the conditions for agriculture improve and the competition with agriculture and the general anthropogenic pressure rises. This is also consistent with a study which found that the Slope Land Conversion program resulted in a significant increase in vegetation cover in the northern Shaanxi Province, but not in the southern part of the province where the climate is more humid [48]. The subsequent fluctuations of the response may also be due to a combination of climatic favorable conditions, anthropogenic pressure, and relatively few data points. The uncertainty of MODIS VCF in estimating tree cover in semi-arid climate should not affect our result, as we mainly included areas with annual precipitation above 400 mm, which also means that we only included small areas with semi-arid climate, mainly in the northwestern part of our study area.

We did not find a significant importance of protected areas on TCC. This could be due to several different things. For example, if a protected area in climatic favorable condition for tree cover has been effective previously, it should already have close to maximum tree cover and thereby tree cover cannot increase further. Whereas areas that have not been protected previously and therefore have experienced extensive deforestation have potential to get relatively high tree cover increase if targeted by afforestation programs. Protected areas also tend to be located in areas that are naturally less likely to be affected by anthropogenic pressure and thereby deforestation [51]. Furthermore, we have relatively few data points for protected areas compared to the size of our overall study area, which might also influence the variable importance result of our random forest models. However, the lack of a detected effect of protected areas might also be a “real result” reflecting limited effectiveness of protected areas. Ineffectiveness of protected areas is a commonly referred problem in China, with many protected areas argued to be so-called “paper parks” [13,19,53], and studies have found high deforestation within protected areas [54].

The RF models do well on the coarser scales, where they explain 41.0% and 47.2% of the variation in tree cover change between 2000 and 2010 (TCC), but the model on the fine scale is only able to explain 8.3% of the variance. The relatively low percentage of variation explained at the fine scale probably reflects stochasticity and local factors, which were not accounted for in our model. Furthermore, the performance of the RF model on the fine scale increased when we excluded cells with low tree cover changes, e.g. cells with a higher uncertainty of an actual increase or decrease of tree cover. The percentage variation explained increased to 11.5% and 13.6%, when only cells with a change of 10% or more or of 15% or more were used, respectively. Nevertheless, the importance rank and the overall relationship of the variables were the same.

Our results show that while there is an overall increase in tree cover within China it is not uniformly distributed. Certain areas, mainly with low or declining population density, low HII, and steep topography, are more likely to experience increase in tree cover and to be protected in the future. Furthermore, even though afforestation and reforestation programs have been partially effective in reducing ecosystem degradation, e.g. soil erosion in some areas [55], they have not been able to reduce the overall problem with soil erosion [56]. In addition, some studies have criticized the afforestation programs for prioritizing economy and wood production [57] rather than ecological restoration, and for not taking local environmental conditions into account [58,59], instead using a “one size fits all” approach. This has resulted in low tree survival rates and little or no restoration effect in many afforestation programs [20,57–60]. Some have even exacerbated the environmental degradation using unsuitable species, which in turn has led to decrease of soil moisture and natural biodiversity [57–60].

Another aspect worth mentioning, is that the increase in tree cover is to a large extent caused by increase in plantations [61], which in many cases consists of monocultures and non-native species [57,59,62]. Monocultures do not contribute to biodiversity in the same degree as natural forest [63–65] and even leads to loss of biodiversity in some cases [66].

In China–and in the world in general–there is a growing recognition and awareness of the importance of forests for the biodiversity and how ecosystem services influence human well-being. Along with the global trend of marginal farmland abandonment [25,67–69], this offers great opportunities for restoration of ecosystems, biodiversity and ecosystems services in the years to come, for example through rewilding [69,70]. Increase in tree cover also offers interesting opportunities for threatened biodiversity. For example if mixed forests are promoted over monocultures, alongside with restoration of degraded habitats and corridors between fragmented habitats of the threatened species, [66,71–74] as an integrated part of the reforestation and afforestation programs.

Other studies have investigated the tree cover change in China locally and also found it is increasing, linked to topography and anthropogenic pressure (e.g. Wang et al. 2016 [25]) This study add to the understanding of tree cover change in China, as it has focus on the entire eastern half of China on both grid cells, county, and prefecture scale. Our results show that areas with an increase in tree cover predominate, especially in central parts of eastern China, as the area with an increase of 10% or more was almost twice as large as the area with a decrease of 10% or more. Furthermore, our findings show that the increase in tree cover in eastern China is associated with low and declining human pressure, climatic condition favoring tree growth, and steep terrain which is less attractive for anthropogenic use, e.g. agriculture. It is important that these associations are taken into account in reforestation and afforestation programs and future studies as they can contribute to more comprehensive predictions and explanations about tree cover change.

## Supporting information

S1 Table Pearson’s correlation coefficient (r) for all variables on all scales. Acronyms: TCC = Tree cover change between 2000 and 2010, CR = Tree cover change rate between 2000 and 2010, AET = Actual evapotranspiration, PopD2000 and PopD2010 = Population density for the year 2000 and 2010, PC00-10 = Population density change between 2000 and 2010, HII = Human Influence Index, GDP/Area = Gross domestic product per area.
(PDF)

S1 Fig Tree cover 2010 for China with study area (selected prefectures). (TIFF)

S2 Fig Maps of the variables for the study area (Fig 1). **a)** Elevation, **b)** slope, **c)** actual evapotranspiration (AET), **d)** population density change between 2000 and 2010 (PC00-10), **e)** Human Influence Index (HII), **F)** Gross domestic product per area (for counties) (GDP/Area).
(TIFF)

S3 Fig Comparison between tree cover change (TCC) and per-year change rate (CR). **a)** 5×5 km grid cells scale, **b)** county scale, and **c)** prefecture scale. The blue lines display LOESS regression fits and are not extrapolated. The red lines display linear regression. In **a)** the blue line lay on top of the red line.
(TIF)

S4 Fig Decrease in mean of squared error in relation to number of trees in the random forest model. **a)** 5×5 km grid cells scale, **b)** county scale, and **c)** prefecture scale. The same trend is seen in all the random forest models for all scales.
(TIF)

S5 Fig **Tree cover change in percent between 2000 and 2010 (TCC) for a) county and b) prefecture scale**. Green colors indicate an increase, beige color indicates a slight increase or decrease and red colors indicate a decrease in tree cover between 2000 and 2010.
(TIF)

S6 Fig Complete partial dependence plot for change in population density and GDP per square kilometer. Complete partial dependence plots of “change in population density (PopD) between 2000 and 2010 for **a)** the prefecture scale, **b)** the 5×5 km grid cells scale, and **c)** the county scale. Complete partial dependence plot of “GDP per square kilometer” for the county scale is showed in **d)**. The ticks inside the graphs indicate the deciles for the data and for all plots are data concentrated closely around 0, e.g. in **b)** only one thick can be seen as all the ticks lay on top of each other. See Figs 4D, 5C, 5E and 6D for cut of plots.
(TIFF)

S7 Fig Prefecture scale. Tree cover change between 2000 and 2010 (TCC) as a function of **a)** slope, **b)** elevation, **c)** actual evapotranspiration, and **d)** change in population density between 2000 and 2010. All are for prefecture scale and **d)** change in population density between 2000 and 2010 is log-modulus transformed. The red lines display LOESS regression fits and are not extrapolated. Blues lines indicate 0 on the y axis.
(TIF)

S8 Fig County scale. Tree cover change between 2000 and 2010 (TCC) as a function of **a)** slope, **b)** elevation, **c)** actual evapotranspiration, **d)** change in population density between 2000 and 2010, and **e)** GDP per square kilometer. All are for county scale and **e)** GDP per square kilometer is log transformed. The red lines display LOESS regression fits and are not extrapolated. Blues lines indicate 0 on the y axis.
(TIFF)

S9 Fig 5×5 km grid cells scale. Tree cover change between 2000 and 2010 (TCC) as a function of **a)** slope, **b)** elevation, **c)** actual evapotranspiration, **d)** change in population density between 2000 and 2010, and **e)** Human Influence Index. All are for the 5×5 km grid cells scale and **d)** change in population density between 2000 and 2010 is log-modulus transformed. The red lines display LOESS regression fits and are not extrapolated. Blues lines indicate 0 on the y axis.
(TIF)

S10 Fig Human Influence Index as a function of slope and elevation for the three scales. Human Influence Index as a function of slope for the **a)** prefecture, **b)** county and **c)** 5×5 km grid cells scale. Human Influence Index as a function of elevation for the **d)** prefecture, **e)** county and **f)** 5×5 km grid cells scale.
(TIF)

## References

[1] FAO. Global Forest Resources Assessment 2015. Food and Agriculture Organization of the United Nations (FAO) <http://www.fao.org>. 2015; 244 pp.

[2] Keenan RJ, Reams GA, Achard F, de Freitas J V., Grainger A, Lindquist E. Dynamics of global forest area: Results from the FAO Global Forest Resources Assessment 2015. Forest Ecology and Management. 2015;352: 9–20.

[3] Sloan S, Sayer JA. Forest Resources Assessment of 2015 shows positive global trends but forest loss and degradation persist in poor tropical countries. Forest Ecology and Management. 2015;352: 134–145.

[4] Hansen MC, Potapov P V., Moore R, Hancher M, Turubanova SA, Tyukavina A, et al High-Resolution Global Maps of 21st-Century Forest Cover Change. Science. 2013;342: 850–853. doi: 10.1126/science.1244693 24233722 10.1126/science.1244693

[5] Vié J-C, Hilton-taylor C, Stuart SN. Wildlife in a Changing World—An Analysis of the 2008 IUCN Red List of Threatened Species. Gland, Switzerland: IUCN 2009.

[6] Aerts R, Honnay O. Forest restoration, biodiversity and ecosystem functioning. BMC Ecology. BioMed Central Ltd; 2011;11: 29 doi: 10.1186/1472-6785-11-29 22115365 10.1186/1472-6785-11-29 PMC3234175

[7] Bonan GB. Forests and climate change: forcings, feedbacks, and the climate benefits of forests. Science. 2008;320: 1444–1449. doi: 10.1126/science.1155121 18556546 10.1126/science.1155121

[8] Miura S, Amacher M, Hofer T, San-Miguel-Ayanz J, Thackway R. Protective functions and ecosystem services of global forests in the past quarter-century. Forest Ecology and Management. 2015;352: 35–46.

[9] FAO. Global Forest Resources Assessment 2010. Food and Agriculture Organization of the United Nations (FAO) <http://www.fao.org>. 2010; 350 pp.

[10] Federici S, Tubiello FN, Salvatore M, Jacobs H, Schmidhuber J. New estimates of CO2 forest emissions and removals: 1990–2015. Forest Ecology and Management. 2015;352: 89–98.

[11] Lewis SL, Lopez-Gonzalez G, Sonke B, Affum-Baffoe K, Baker TR, Ojo LO, et al Increasing carbon storage in intact African tropical forests. Nature. 2009;457: 1003–1006. doi: 10.1038/nature07771 19225523 10.1038/nature07771

[12] Liu J, Diamond J. China’s environment in a globalizing world. Nature. 2005;435: 1179–1186. doi: 10.1038/4351179a 15988514 10.1038/4351179a

[13] Harkness J. Recent Trends in Forestry and Conservation of Biodiversity in China. The China Quarterly. Cambridge University Press; 1998;156: 911.

[14] Yin R, Xu J, Li Z, Liu C. China’s Ecological Rehabilitation: The Unprecedented Efforts and Dramatic Impacts of Reforestation and Slope Protection in Western China. China Environment Series. 2005;7: 17–32.

[15] Sandel B, Svenning J-C. Human impacts drive a global topographic signature in tree cover. Nature communications. Nature Publishing Group; 2013;4: 1–7. 10.1038/ncomms3474 24064801

[16] Odgaard M V., Bøcher PK, Dalgaard T, Moeslund JE, Svenning JC. Human-driven topographic effects on the distribution of forest in a flat, lowland agricultural region. Journal of Geographical Sciences. 2014;24: 76–92.

[17] Liang G, Ding S. Driving factors of forest landscape change in Yiluo River basin. Journal of Geographical Sciences. 2006;16: 415–422.

[18] Wu R, Zhang S, Yu DW, Zhao P, Li X, Wang L, et al Effectiveness of China’s nature reserves in representing ecological diversity. Frontiers in Ecology and the Environment. 2011;9: 383–389.

[19] Jim CY, Xu SSW. Recent protected-area designation in China: An evaluation of administrative and statutory procedures. Geographical Journal. 2004;170: 39–50.

[20] Wang XM, Zhang CX, Hasi E, Dong ZB. Has the Three Norths Forest Shelterbelt Program solved the desertification and dust storm problems in arid and semiarid China? Journal of Arid Environments. 2010;74: 13–22.

[21] Zhang Y, Song C. Impacts of afforestation, deforestation, and reforestation on forest cover in China from 1949 to 2003. Journal of Forestry. Society of American Foresters; 2006;104: 383–387.

[22] Wenhua L. Degradation and restoration of forest ecosystems in China. Forest Ecology and Management. 2004;201: 33–41.

[23] Wang G, Innes JL, Lei J, Dai S, Wu SW. China’s Forestry Reforms. Science. 2007;318: 1556–1557. doi: 10.1126/science.1147247 18063773 10.1126/science.1147247

[24] Cao G-Y, Chen G, Pang L-H, Zheng X-Y, Nilsson S. Urban growth in China: past, prospect, and its impacts. Population & Environment. 2012;33: 137–160.

[25] Wang C, Gao Q, Wang X, Yu M, Malhi Y, Gardner TA, et al Spatially differentiated trends in urbanization, agricultural land abandonment and reclamation, and woodland recovery in Northern China. Scientific Reports. Nature Publishing Group; 2016;6: 37658 doi: 10.1038/srep37658 27874092 10.1038/srep37658 PMC5118683

[26] Du S, Shi P, Rompaey A. The Relationship between Urban Sprawl and Farmland Displacement in the Pearl River Delta, China. Land. 2013;3: 34–51.

[27] DiMiceli CM, Carroll ML, Sohlberg RA, Huang C, Hansen MC, Townshend JRG. Annual Global Automated MODIS Vegetation Continuous Fields (MOD44B) at 250 m Spatial Resolution for Data Years Beginning Day 65, 2000–2010, Collection 5. University of Maryland, College Park, MD, USA. Percent Tree Cover. University of Maryland, College Park, MD, USA. 2011;

[28] MODIS Land Team. Status for: Vegetation Continuous Fields (MOD44)—General Accuracy Statement. 2016; Available at: https://landval.gsfc.nasa.gov/ProductStatus.php?ProductID=MOD44

[29] Hansen MC, DeFries RS, Townshend JRG, Carroll M, Dimiceli C, Sohlberg RA. Global Percent Tree Cover at a Spatial Resolution of 500 Meters: First Results of the MODIS Vegetation Continuous Fields Algorithm. Earth Interactions. 2003;7: 1–15.

[30] Townshend JRG, Hansen MC, Carroll M, DiMiceli C, Sohlberg R, Huang C. User Guide for the MODIS Vegetation Continuous Fields product Collection 5 version 1. 2011; Available at: https://lpdaac.usgs.gov/dataset_discovery/modis/modis_products_table/mod44b.

[31] Liu X, Liu H, Qiu S, Wu X, Tian Y, Hao Q. An Improved Estimation of Regional Fractional Woody/Herbaceous Cover Using Combined Satellite Data and High-Quality Training Samples. Remote Sensing. 2017;9: 32.

[32] Jarvis A, Reuter HI, Nelson A, Guevara E. Hole-filled SRTM for the globe Version 4, available from the CGIAR-CSI SRTM 90m Database. Available at: http://srtm.csi.cgiar.org. 2008.

[33] Trabucco A, Zomer RJ. Global High-Resolution Soil-Water Balance Geospatial Database. CGIAR Consortium for Spatial Information. Available from the CGIAR-CSI GeoPortal at: http://www.csi.cgiar.org/. 2010.

[34] Center for International Earth Science Information Network—CIESIN—Columbia University. Gridded Population of the World, Version 4 (GPWv4): Population Density Adjusted to Match 2015 Revision of UN WPP Country Totals. Palisades. NY: NASA Socioeconomic Data and Applications Center (SEDAC); 2015.

[35] Wildlife Conservation Society—WCS—and Center for Interntional Earth Science Infrmation Network—CIESIN—Columbia University. Last of the Wild Project, Version 2, 2005 (LWP-2): Global Human Influence Index (HII) Dataset (IGHP). Palisades, NY: NASA Socioeconomic Data and Applications Center (SEDAC).

[36] National Bureau of Statistics of China. 2001 China Statistical Yearbook for Regional Economy. China Statistics Press; 2001.

[37] IUCN, UNEP-WCMC. The World Database on Protected Areas (WDPA). Cambridge, UK: UNEP-WCMC Accessed 16 January 2015. Available at: www.protectedplanet.net. 2014.

[38] Liaw A, Wiener M. Classification and regression by randomForest. R news. 2002;

[39] Breiman L. Random forests. Machine Learning. Kluwer Academic Publishers; 2001;45: 5–32.

[40] Prasad AM, Iverson LR, Liaw A. Newer classification and regression tree techniques: Bagging and random forests for ecological prediction. Ecosystems. 2006;9: 181–199.

[41] Lawler JJ, White D, Neilson RP, Blaustein AR. Predicting climate-induced range shifts: Model differences and model reliability. Global Change Biology. 2006;12: 1568–1584.

[42] Li X, Wang Y. Applying various algorithms for species distribution modelling. Integrative Zoology. 2013;8: 124–135. doi: 10.1111/1749-4877.12000 23731809 10.1111/1749-4877.12000

[43] Díaz-Uriarte R, Alvarez de Andrés S. Gene selection and classification of microarray data using random forest. BMC bioinformatics. 2006;7: 3 doi: 10.1186/1471-2105-7-3 16398926 10.1186/1471-2105-7-3 PMC1363357

[44] Grömping U. Variable Importance Assessment in Regression: Linear Regression versus Random Forest. The American Statistician. 2009;63: 308–319.

[45] Altmann A, Tolosi L, Sander O, Lengauer T. Permutation importance: A corrected feature importance measure. Bioinformatics. 2010;26: 1340–1347. doi: 10.1093/bioinformatics/btq134 20385727 10.1093/bioinformatics/btq134

[46] Ender C. vita: Variable Importance Testing Approaches. 2015.

[47] Gregorutti B, Michel B, Saint-Pierre P. Correlation and variable importance in random forests. Statistics and Computing. 2016; 1–20.

[48] Zhou H, Van Rompaey A, Wang J. Detecting the impact of the “Grain for Green” program on the mean annual vegetation cover in the Shaanxi province, China using SPOT-VGT NDVI data. Land Use Policy. 2009;26: 954–960.

[49] Acácio V, Holmgren M, Moreira F, Mohren GMJ. Oak persistence in Mediterranean landscapes: The combined role of management, topography, and wildfires. Ecology and Society. 2010;15.

[50] Scott JM, Davis FW, Mcghie RG, Wright RG, Estes J, Scott JM, et al Nature Reserves: Do They Capture the Full Range of America’ s Biological Diversity? Ecological Applications. 2001;11: 999–1007.

[51] Joppa LN, Pfaff A. High and far: Biases in the location of protected areas. PLoS ONE. 2009;4: 1–6. 10.1371/journal.pone.0008273 PMC2788247 20011603

[52] Rouget M, Richardson DM, Cowling RM. The current configuration of protected areas in the Cape Floristic Region, South Africa—Reservation bias and representation of biodiversity patterns and processes. Biological Conservation. 2003;112: 129–145.

[53] Jim CY, Xu, Shaowei S. Getting Out of the Woods: Quandaries of Protected Area Management in China. Mountain Research and Development. 2003;23: 222–226.

[54] Chen H, Yi ZF, Schmidt-Vogt D, Ahrends A, Beckschäfer P, Kleinn C, et al Pushing the limits: The pattern and dynamics of rubber monoculture expansion in Xishuangbanna, SW China. PLoS ONE. 2016;11: 1–15. 10.1371/journal.pone.0150062 PMC4764337 26907479

[55] Deng L, Shangguan Z, Li R. Effects of the grain-for-green program on soil erosion in China. International Journal of Sediment Research. 2012;27: 120–127.

[56] Wang X, Zhao X, Zhang Z, Yi L, Zuo L, Wen Q, et al Assessment of soil erosion change and its relationships with land use/cover change in China from the end of the 1980s to 2010. CATENA. 2016;137: 256–268.

[57] Cao S. Why large-scale afforestation efforts in China have failed to solve the desertification problem. Environmental Science and Technology. 2008;42: 1826–1831. 18409601 10.1021/es0870597

[58] Cao S, Chen L, Shankman D, Wang C, Wang X, Zhang H. Excessive reliance on afforestation in China’s arid and semi-arid regions: Lessons in ecological restoration. Earth-Science Reviews. 2011;104: 240–245.

[59] Cao S, Sun G, Zhang Z, Chen L, Feng Q, Fu B, et al Greening China naturally. Ambio. 2011;40: 828–831. doi: 10.1007/s13280-011-0150-8 22338721 10.1007/s13280-011-0150-8 PMC3357754

[60] Cao S, Tian T, Chen L, Dong X, Yu X, Wang G. Damage caused to the environment by reforestation policies in arid and semi-arid areas of China. Ambio. 2010;39: 279–283. doi: 10.1007/s13280-010-0038-z 20799677 10.1007/s13280-010-0038-z PMC3357704

[61] FAO (Food and Agriculture Organization of the United Nations). China—Global Forest Resources Assessment 2015 –Country Report. 2015.

[62] Liu J, Li S, Ouyang Z, Tam C, Chen X. Ecological and socioeconomic effects of China’s policies for ecosystem services. Proceedings of the National Academy of Sciences of the United States of America. 2008;105: 9477–9482. doi: 10.1073/pnas.0706436105 18621700 10.1073/pnas.0706436105 PMC2474515

[63] Bremer LL, Farley KA. Does plantation forestry restore biodiversity or create green deserts? A synthesis of the effects of land-use transitions on plant species richness. Biodiversity and Conservation. 2010;19: 3893–3915.

[64] Sayer J, Chokkalingam U, Poulsen J. The restoration of forest biodiversity and ecological values. Forest Ecology and Management. 2004;201: 3–11.

[65] Brockerhoff EG, Jactel H, Parrotta JA, Quine CP, Sayer J. Plantation forests and biodiversity: Oxymoron or opportunity? Biodiversity and Conservation. 2008;17: 925–951.

[66] Hua F, Wang X, Zheng X, Fisher B, Wang L, Zhu J, et al Opportunities for biodiversity gains under the world’s largest reforestation programme. Nature Communications. Nature Publishing Group; 2016;7: 12717 doi: 10.1038/ncomms12717 27598524 10.1038/ncomms12717 PMC5025860

[67] Keenleyside C, Tucker G. Farmland abandonment in the EU: an assessment of trends and prospects. 2010; 93.

[68] Queiroz C, Beilin R, Folke C, Lindborg R. Farmland abandonment: Threat or opportunity for biodiversity conservation? A global review. Frontiers in Ecology and the Environment. 2014;12: 288–296.

[69] Ceaușu S, Hofmann M, Navarro LM, Carver S, Verburg PH, Pereira HM. Mapping opportunities and challenges for rewilding in Europe. Conservation Biology. 2015;29: 1017–1027. doi: 10.1111/cobi.12533 25997361 10.1111/cobi.12533 PMC4584510

[70] Navarro LM, Pereira HM. Rewilding abandoned landscapes in Europe. Rewilding European Landscapes. 2015; 3–23.

[71] Qin Y, Nyhus PJ, Larson CL, Carroll CJW, Muntifering J, Dahmer TD, et al An assessment of South China tiger reintroduction potential in Hupingshan and Houhe National Nature Reserves, China. Biological Conservation. 2015;182: 72–86.

[72] Li L, Yu S, Ren B, Li M, Wu R, Long Y. A study on the carrying capacity of the available habitat for the Rhinopithecus bieti population at Mt. Laojun in Yunnan, China. Environmental Science and Pollution Research. 2009/03/25. 2009;16: 474–478. doi: 10.1007/s11356-009-0130-8 19308474 10.1007/s11356-009-0130-8

[73] IUCN/SSC. Guidelines for Reintroductions and Other Conservation Translocations. Version 1.0. Gland, Switzerland: IUCN Species Survival Commission 2013.

[74] Wang F, McShea WJ, Wang D, Li S, Zhao Q, Wang H, et al Evaluating Landscape Options for Corridor Restoration between Giant Panda Reserves. PloS one. 2014;9: e105086 doi: 10.1371/journal.pone.0105086 25133757 10.1371/journal.pone.0105086 PMC4136856

