# Supplementary material for: Recent tree cover increases in eastern China linked to low, declining human pressure, steep topography, and climatic conditions favoring tree growth
Source: PLoS One. 2017 Jun 7;12(6):e0177552. doi: 10.1371/journal.pone.0177552 (PMC5462372; doi:10.1371/journal.pone.0177552)
Supplement: S1 Table — Acronyms: TCC = Tree cover change between 2000 and 2010, CR = Tree cover change rate between 2000 and 2010, AET = Actual evapotranspiration, PopD2000 and PopD2010 = Population density for the year 2000 and 2010, PC00-10 = Population density change between 2000 and 2010, HII = Human Influence Index, GDP/Area = Gross domestic product per area. (PDF) [file pone.0177552.s001.pdf]

		TCC	CR	AET	Elevation	Slope	PopD 2000	PopD 2010	PC 00-10	HII	GDP/ Area
**5×5 km**	TCC		0.82	0.08	0.03	0.17	-0.02	-0.02	-0.01	-0.05	
	CR	0.82		0.08	0.06	0.19	-0.03	-0.03	-0.02	-0.06	
	AET	0.08	0.08		-0.24	0.16	0.07	0.07	0.03	0.04	
	Elevation	0.03	0.06	-0.24		0.56	-0.16	-0.15	-0.05	-0.49	
	Slope	0.17	0.19	0.16	0.56		-0.16	-0.15	-0.06	-0.41	
	PopD2000	-0.02	-0.03	0.07	-0.16	-0.16		0.99	0.64	0.33	
	PopD2010	-0.02	-0.03	0.07	-0.15	-0.15	0.99		0.74	0.32	
	PC00-10	-0.01	-0.02	0.03	-0.05	-0.06	0.64	0.74		0.17	
	HII	-0.05	-0.06	0.04	-0.49	-0.41	0.33	0.32	0.17		
**County**	TCC		0.96	0.10	0.19	0.39	-0.17	-0.16	-0.08	-0.26	-0.11
	CR	0.96		0.07	0.26	0.43	-0.18	-0.17	-0.10	-0.26	-0.19
	AET	0.10	0.07		-0.18	0.24	0.04	0.05	0.06	-0.15	0.05
	Elevation	0.19	0.26	-0.18		0.73	-0.24	-0.22	-0.10	-0.54	-0.16
	Slope	0.39	0.43	0.24	0.73		-0.31	-0.28	-0.12	-0.65	-0.18
	PopD2000	-0.17	-0.18	0.04	-0.24	-0.31		0.99	0.74	0.65	0.52
	PopD2010	-0.16	-0.17	0.05	-0.22	-0.28	0.99		0.83	0.63	0.51
	PC00-10	-0.08	-0.10	0.06	-0.10	-0.12	0.74	0.83		0.38	0.38
	HII	-0.26	-0.26	-0.15	-0.54	-0.65	0.65	0.63	0.38		0.40
	GDP/Area	-0.11	-0.19	0.05	-0.16	-0.18	0.52	0.51	0.38	0.40	
**Prefecture**	TCC		0.80	0.11	0.25	0.48	-0.26	-0.24	-0.11	-0.27	-0.07
	CR	0.80		0.06	0.32	0.51	-0.24	-0.22	-0.13	-0.24	-0.06
	AET	0.11	0.06		-0.24	0.19	0.19	0.19	0.14	-0.10	0.10
	Elevation	0.25	0.32	-0.24		0.75	-0.41	-0.36	-0.14	-0.6	-0.11
	Slope	0.48	0.51	0.19	0.75		-0.45	-0.39	-0.13	-0.70	-0.09
	PopD2000	-0.26	-0.24	0.19	-0.41	-0.45		0.98	0.72	0.73	0.25
	PopD2010	-0.24	-0.22	0.19	-0.36	-0.39	0.98		0.84	0.67	0.27
	PC00-10	-0.11	-0.13	0.14	-0.14	-0.13	0.72	0.84		0.37	0.27
	HII	-0.27	-0.24	-0.10	-0.60	-0.70	0.73	0.67	0.37		0.25
	GDP/Area	-0.07	-0.06	0.10	-0.11	-0.09	0.25	0.27	0.27	0.25	
