# Supplementary figures and images for: Recent tree cover increases in eastern China linked to low, declining human pressure, steep topography, and climatic conditions favoring tree growth
Source: PLoS One. 2017 Jun 7;12(6):e0177552. doi: 10.1371/journal.pone.0177552 (PMC5462372; doi:10.1371/journal.pone.0177552)

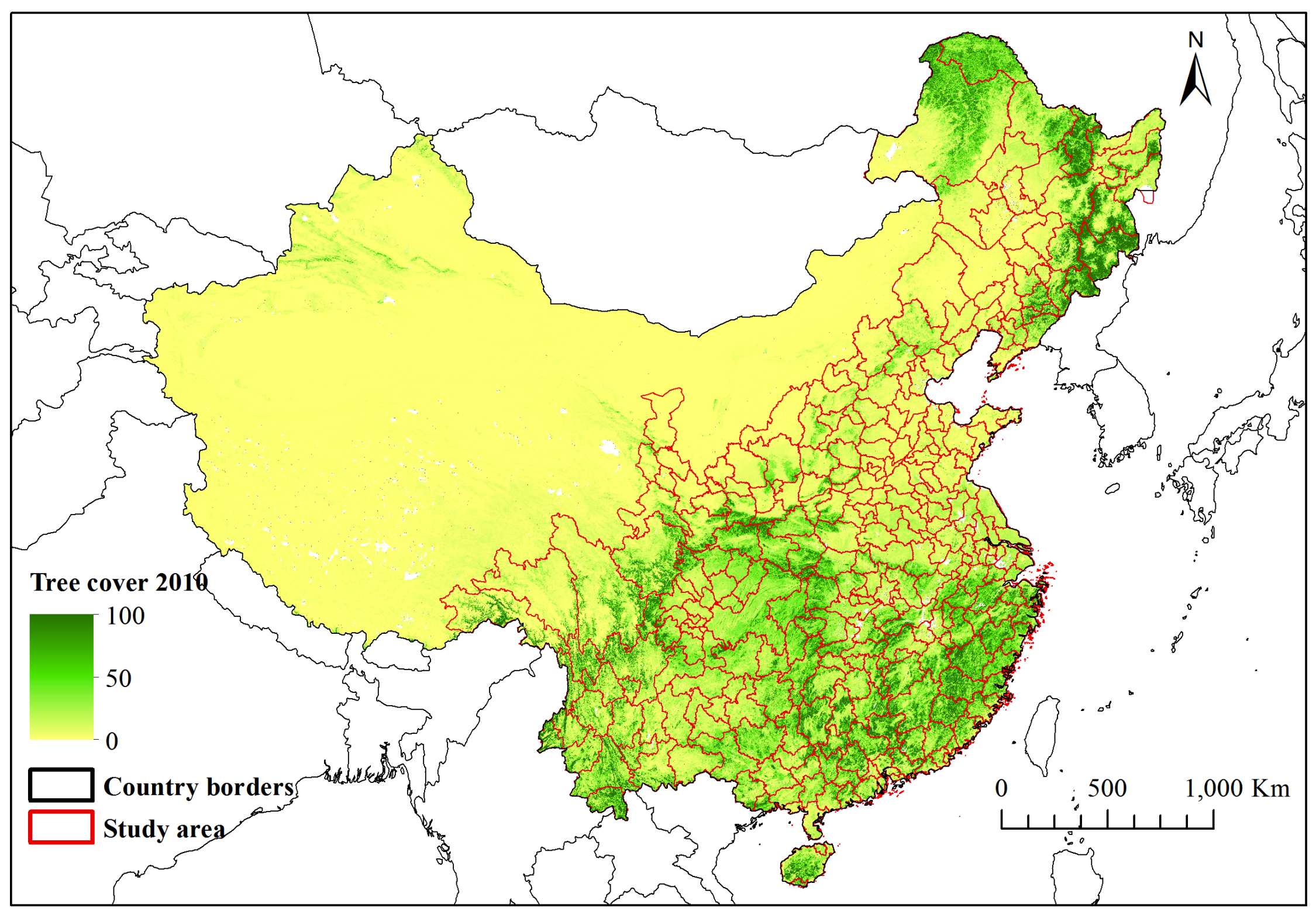

Supplement: S1 Fig — (TIFF) [file pone.0177552.s002.tiff]

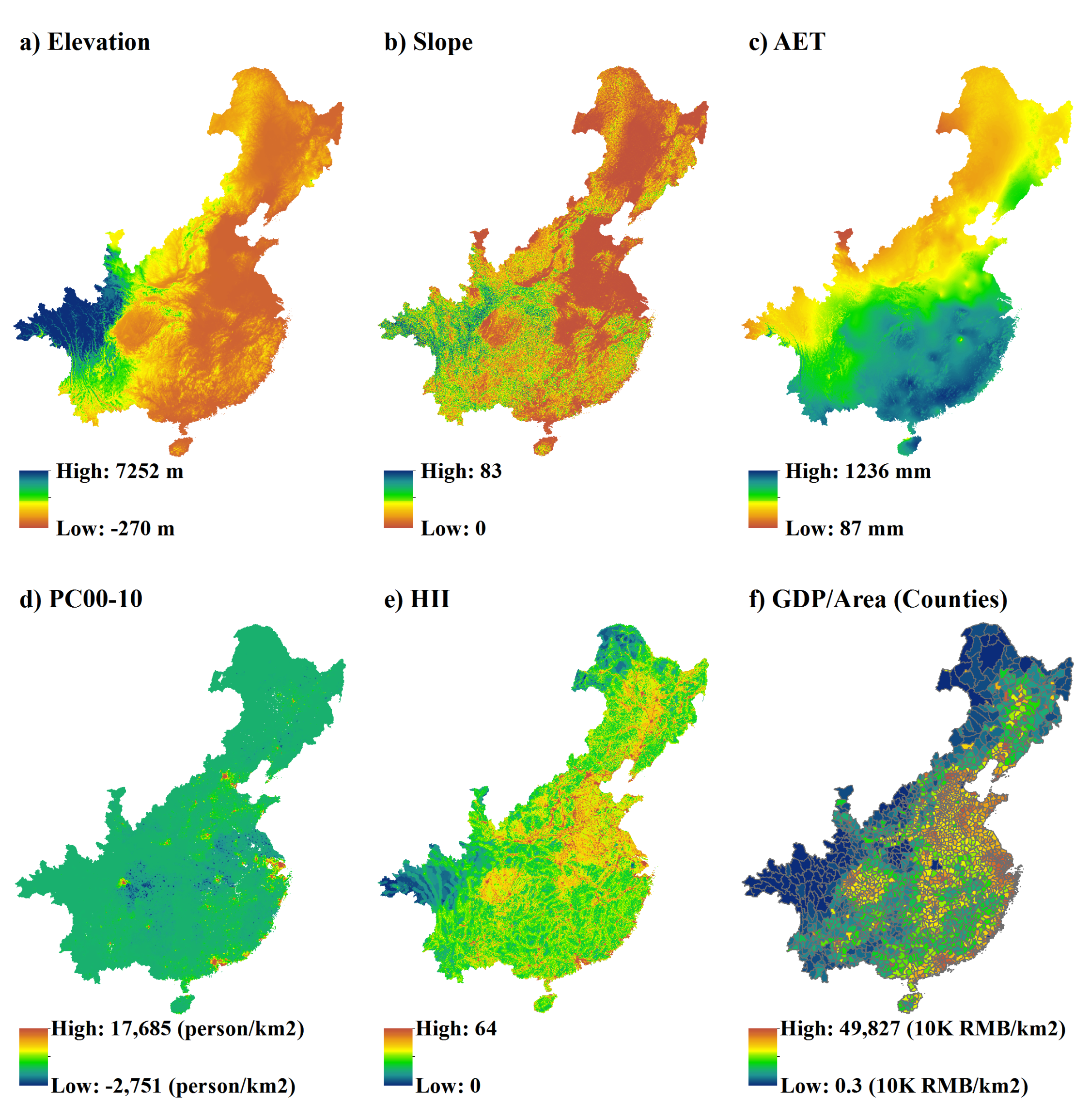

Supplement: S2 Fig — a) Elevation, b) slope, c) actual evapotranspiration (AET), d) population density change between 2000 and 2010 (PC00-10), e) Human Influence Index (HII), F) Gross domestic product per area (for counties) (GDP/Area). (TIFF) [file pone.0177552.s003.tiff]

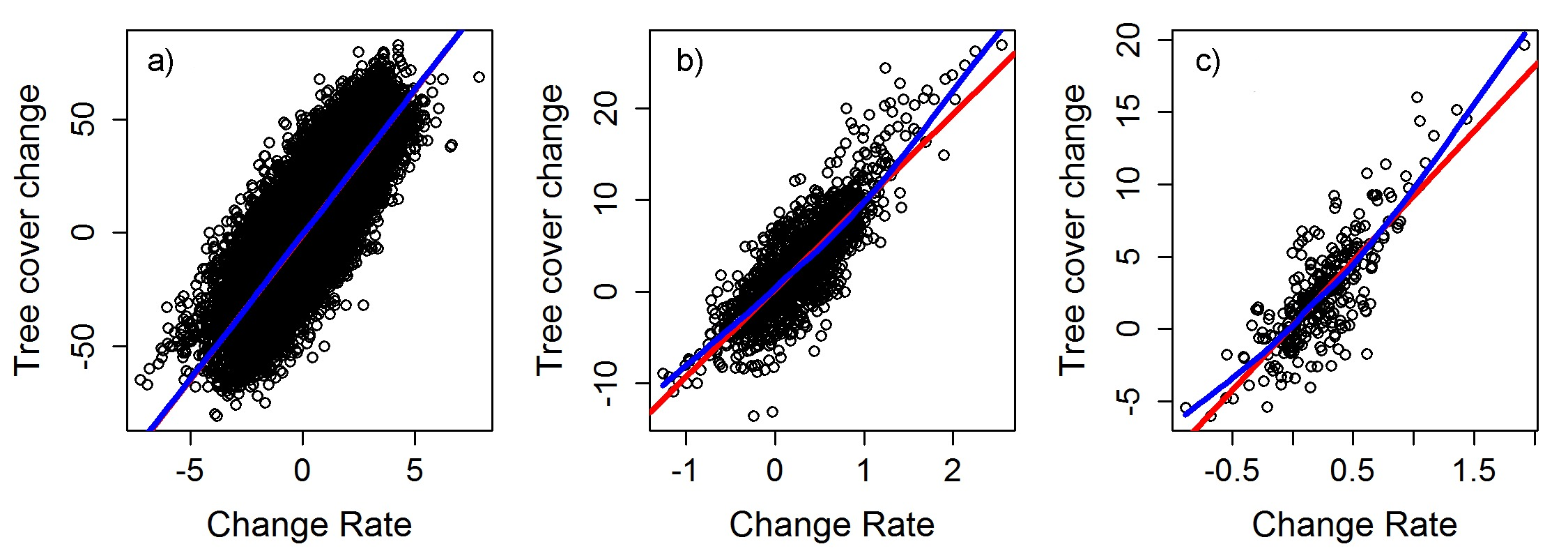

Supplement: S3 Fig — a) 5×5 km grid cells scale, b) county scale, and c) prefecture scale. The blue lines display LOESS regression fits and are not extrapolated. The red lines display linear regression. In a) the blue line lay on top of the red line. (TIF) [file pone.0177552.s004.tif]

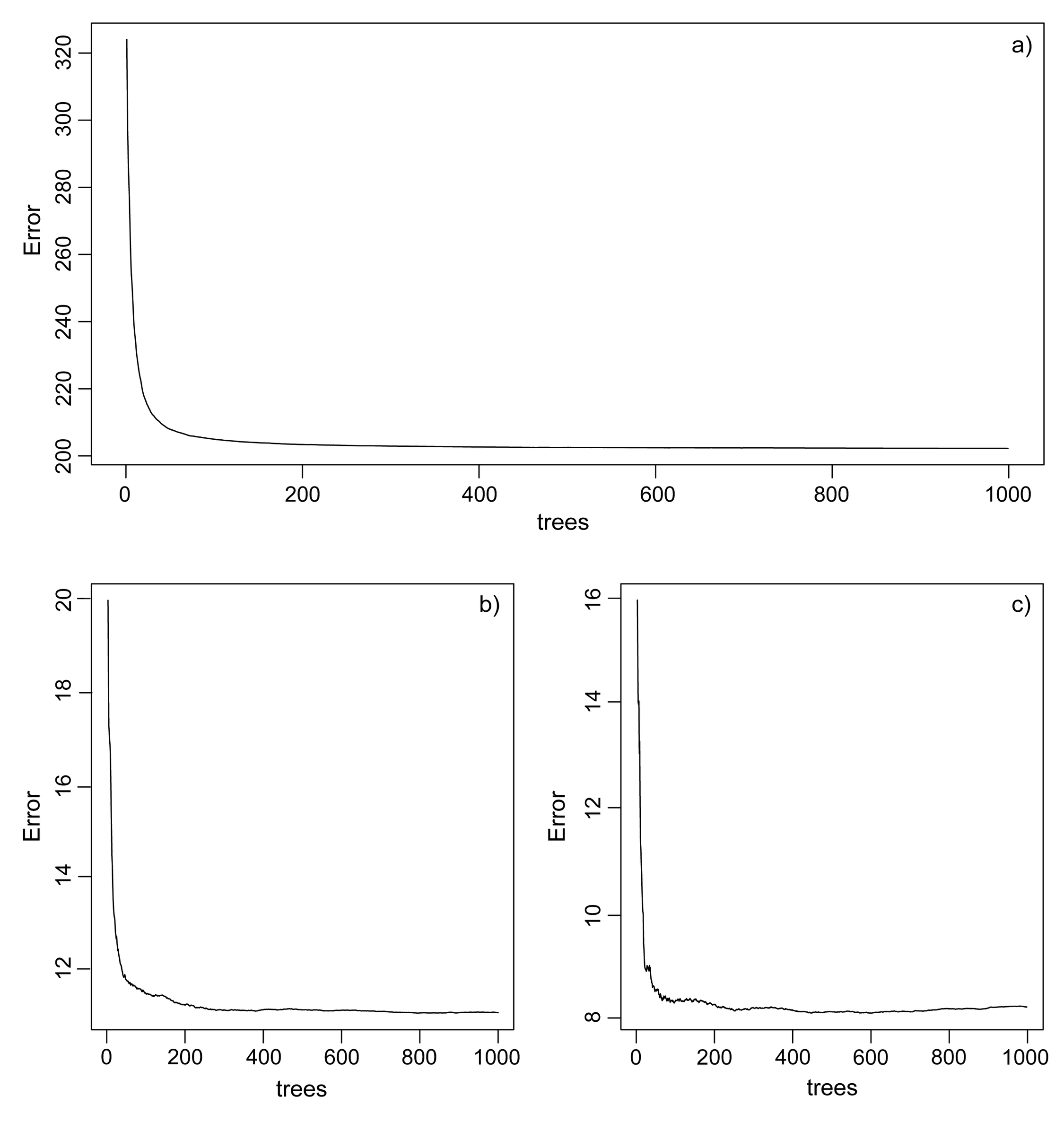

Supplement: S4 Fig — a) 5×5 km grid cells scale, b) county scale, and c) prefecture scale. The same trend is seen in all the random forest models for all scales. (TIF) [file pone.0177552.s005.tif]

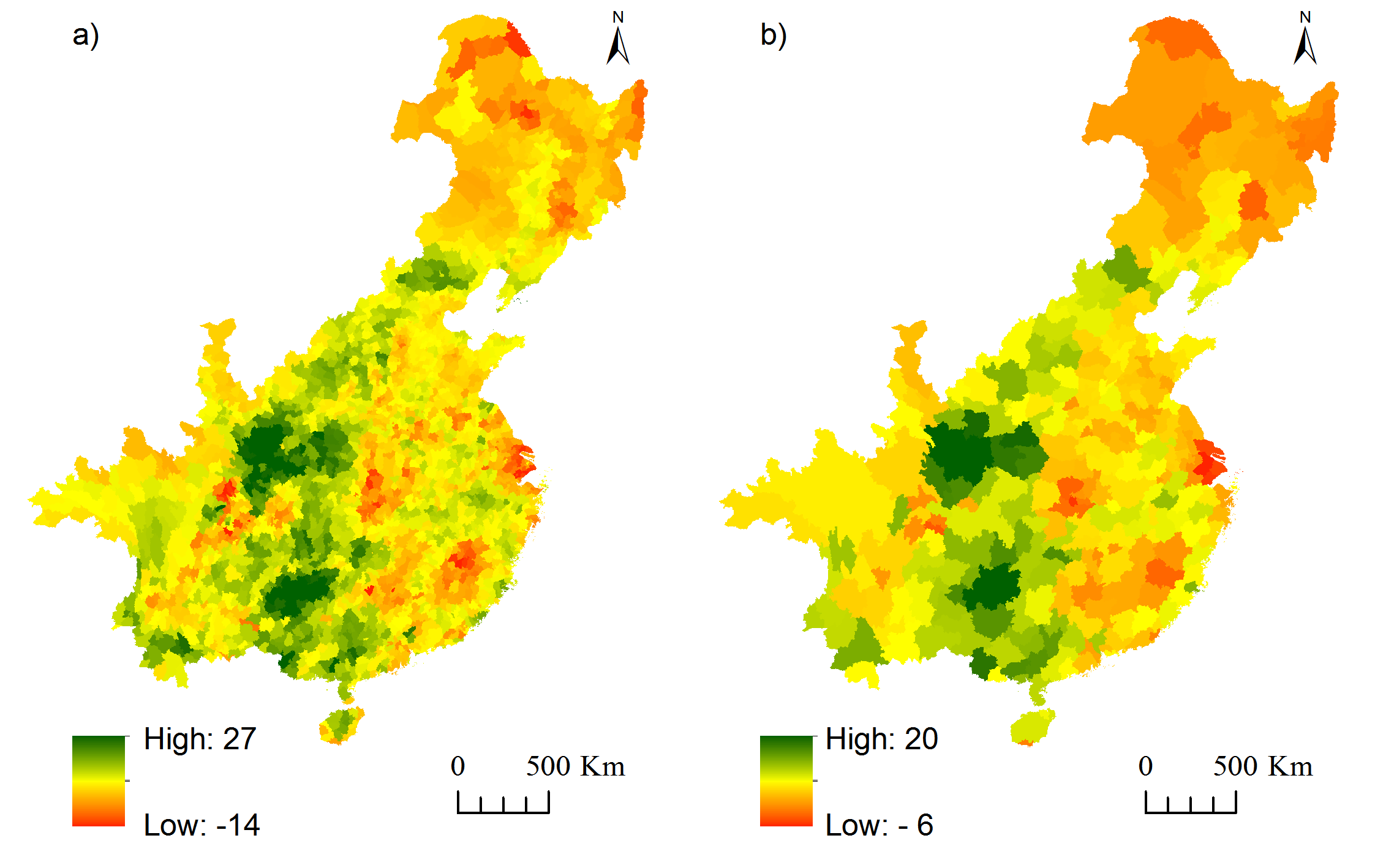

Supplement: S5 Fig — Tree cover change in percent between 2000 and 2010 (TCC) for a) county and b) prefecture scale. Green colors indicate an increase, beige color indicates a slight increase or decrease and red colors indicate a decrease in tree cover between 2000 and 2010. (TIF) [file pone.0177552.s006.tif]

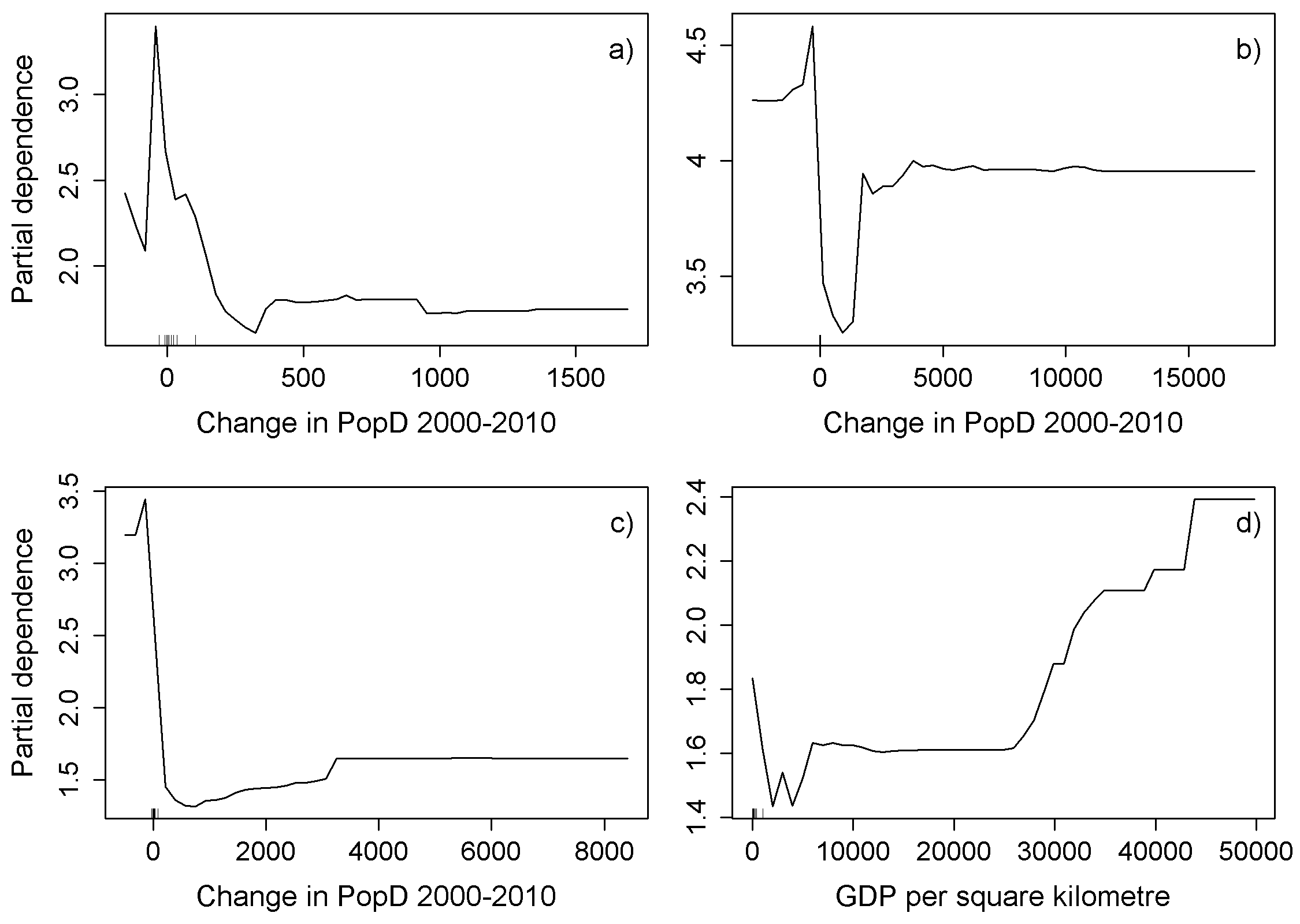

Supplement: S6 Fig — Complete partial dependence plots of “change in population density (PopD) between 2000 and 2010 for a) the prefecture scale, b) the 5×5 km grid cells scale, and c) the county scale. Complete partial dependence plot of “GDP per square kilometer” for the county scale is showed in d). The ticks inside the graphs indicate the deciles for the data and for all plots are data concentrated closely around 0, e.g. in b) only one thick can be seen as all the ticks lay on top of each other. See Figs 4D, 5C, 5E and 6D for cut of plots. (TIFF) [file pone.0177552.s007.tiff]

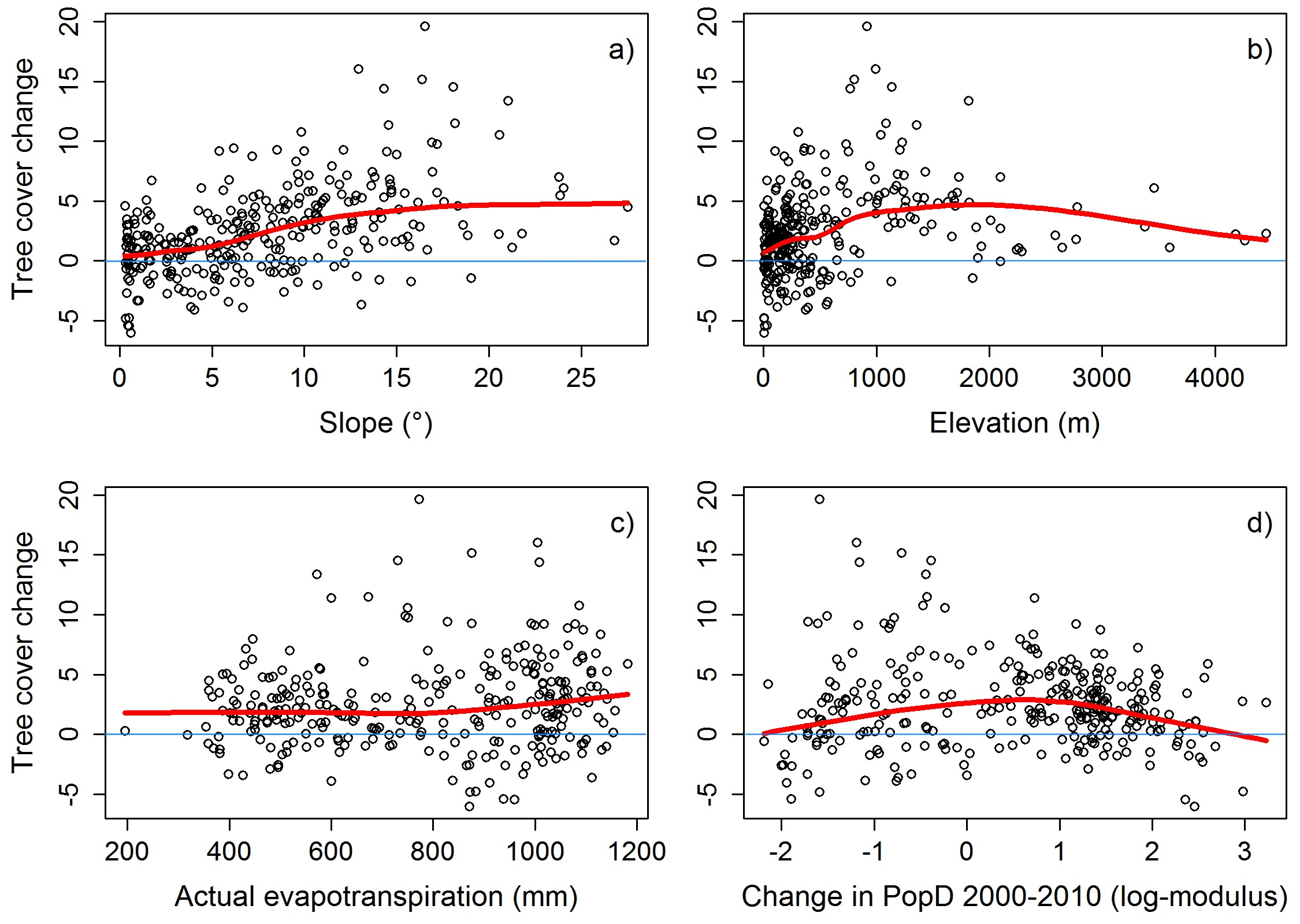

Supplement: S7 Fig — Tree cover change between 2000 and 2010 (TCC) as a function of a) slope, b) elevation, c) actual evapotranspiration, and d) change in population density between 2000 and 2010. All are for prefecture scale and d) change in population density between 2000 and 2010 is log-modulus transformed. The red lines display LOESS regression fits and are not extrapolated. Blues lines indicate 0 on the y axis. (TIF) [file pone.0177552.s008.tif]

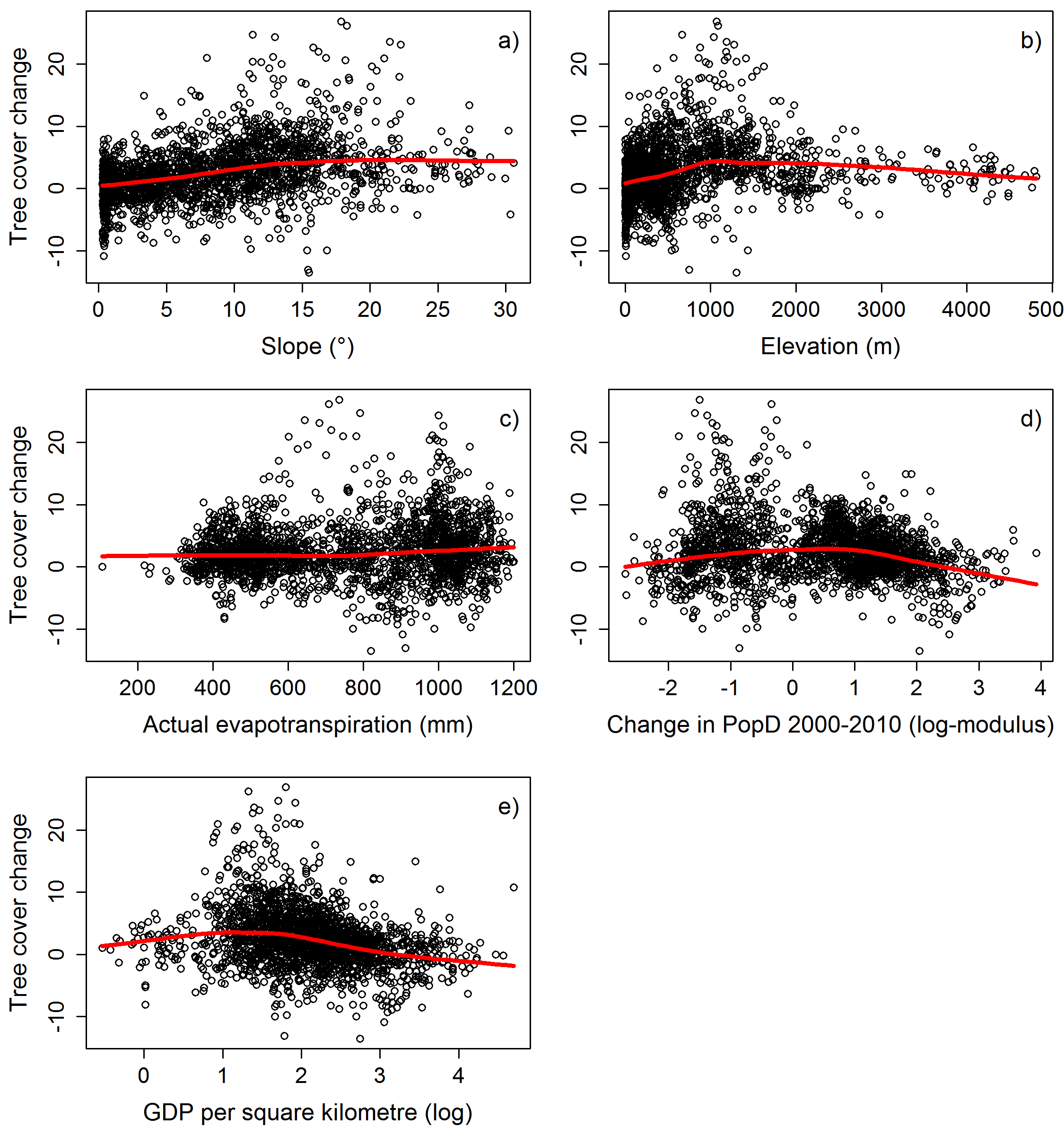

Supplement: S8 Fig — Tree cover change between 2000 and 2010 (TCC) as a function of a) slope, b) elevation, c) actual evapotranspiration, d) change in population density between 2000 and 2010, and e) GDP per square kilometer. All are for county scale and e) GDP per square kilometer is log transformed. The red lines display LOESS regression fits and are not extrapolated. Blues lines indicate 0 on the y axis. (TIFF) [file pone.0177552.s009.tiff]

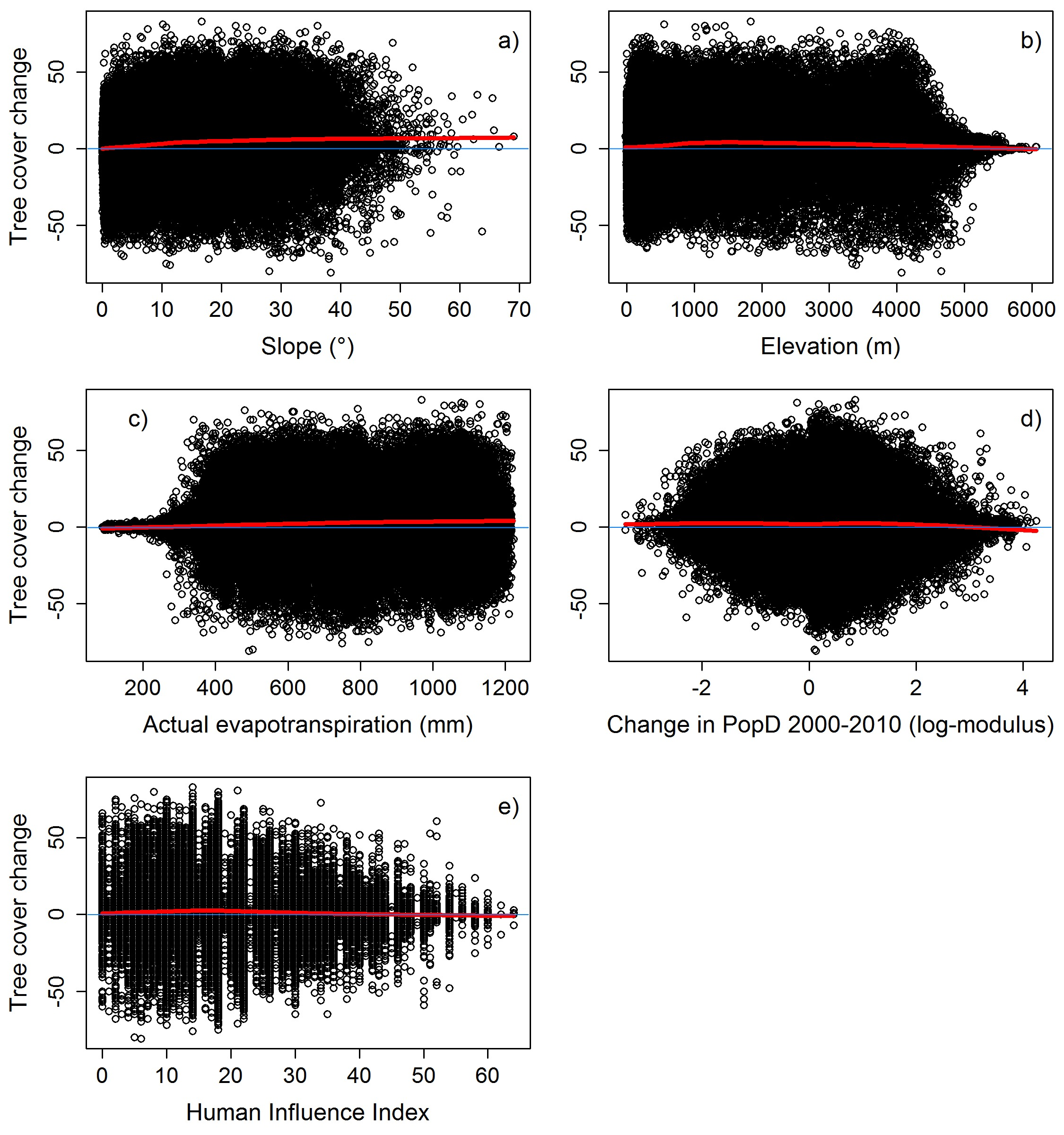

Supplement: S9 Fig — Tree cover change between 2000 and 2010 (TCC) as a function of a) slope, b) elevation, c) actual evapotranspiration, d) change in population density between 2000 and 2010, and e) Human Influence Index. All are for the 5×5 km grid cells scale and d) change in population density between 2000 and 2010 is log-modulus transformed. The red lines display LOESS regression fits and are not extrapolated. Blues lines indicate 0 on the y axis. (TIF) [file pone.0177552.s010.tif]

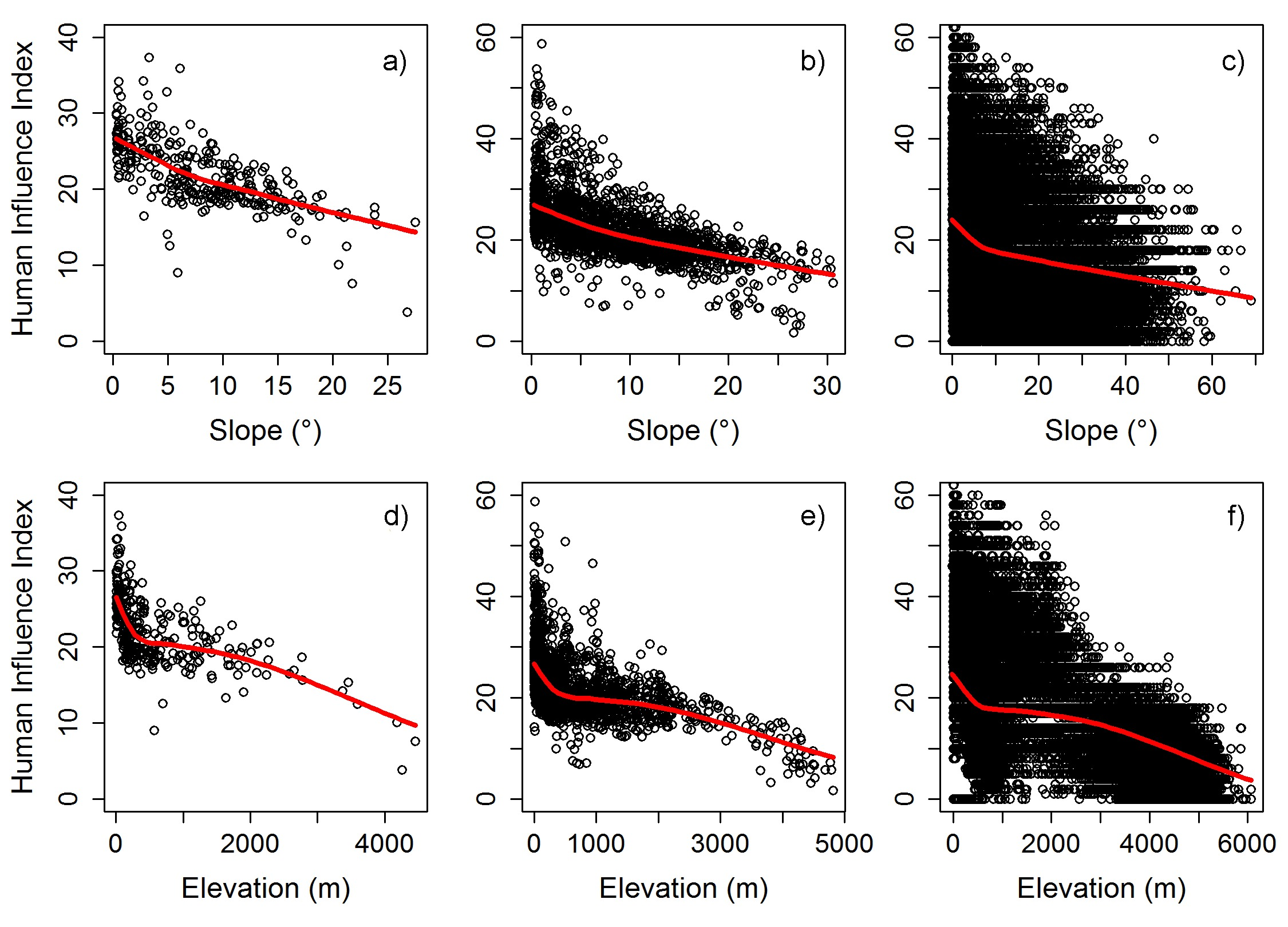

Supplement: S10 Fig — Human Influence Index as a function of slope for the a) prefecture, b) county and c) 5×5 km grid cells scale. Human Influence Index as a function of elevation for the d) prefecture, e) county and f) 5×5 km grid cells scale. (TIF) [file pone.0177552.s011.tif]
